# The mitotic checkpoint kinase BUB1 is a direct and actionable target of MYB in adenoid cystic carcinoma

**DOI:** 10.1002/1873-3468.14786

**Published:** 2023-12-27

**Authors:** Ylenia Cicirò, Denise Ragusa, Paloma Tejera Nevado, Rossano Lattanzio, Gianluca Sala, Tessa DesRochers, Melissa Millard, Mattias K. Andersson, Göran Stenman, Arturo Sala

**Affiliations:** ^1^ Department of Life Sciences, Centre for Inflammation Research and Molecular Medicine (CIRTM) Brunel University London Uxbridge UK; ^2^ Department of Life Sciences, Centre for Genomic Engineering and Maintenance (CenGEM) Brunel University London Uxbridge UK; ^3^ Sahlgrenska Center for Cancer Research Department of Pathology University of Gothenburg Sweden; ^4^ Center for Advanced Studies and Technology (CAST); Department of Innovative Technologies in Medicine & Dentistry University of Chieti‐Pescara Italy; ^5^ Kiyatec Greenville SC USA

**Keywords:** 3D assay, BAY1816032, ChIP sequencing, head and neck cancer, PDX

## Abstract

Adenoid cystic carcinoma (ACC) is a head and neck cancer that frequently originates in salivary glands, but can also strike other exocrine glands such as the breast. A key molecular alteration found in the majority of ACC cases is *MYB* gene rearrangements, leading to activation of the oncogenic transcription factor MYB. In this study, we used immortalised breast epithelial cells and an inducible *MYB* transgene as a model of ACC. Molecular profiling confirmed that MYB‐driven gene expression causes a transition into an ACC‐like state. Using this new cell model, we identified BUB1 as a targetable kinase directly controlled by MYB, whose pharmacological inhibition caused MYB‐dependent synthetic lethality, growth arrest and apoptosis of patient‐derived cells and organoids.

## Graphical abstract

MYB is a transcription factor altered in adenoid cystic carcinoma (ACC), a deadly cancer originating in exocrine glands. Here, we show that ectopic expression of MYB in breast epithelial cells induces an ACC‐like state and transcriptional activation of the *BUB1* kinase gene. Pharmacological targeting of the BUB1 kinase by a small molecule inhibitor discloses a vulnerability of MYB‐expressing ACC.

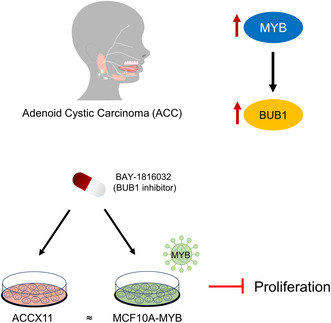

## Abbreviations

**ACC**, adenoid cystic carcinoma

**ChIP**, chromatin immunoprecipitation

**ChIP‐seq**, chromatin immunoprecipitation sequencing

**DAB**, Diaminobenzidine

**DOX**, doxycycline

**EV**, empty vector

**FDR**, false discovery rate

**GFP**, green fluorescent protein

**GO**, gene ontology

**GSEA**, Gene Set Enrichment Analysis

**hEGF**, human epidermal growth factor

**MACS**, Model‐based Analysis of ChIP‐Seq

**MBS**, MYB binding site

**NES**, Normalised Enrichment Score

**NSG**, normal salivary glands

***P* ADJ**, *P* value adjusted

**PDX**, patient derived xenograft

**PenStrep**, penicillin–streptomycin

**RLU**, Relative Light Unit

**RMA**, Robust Multi‐Array Average

**RNA‐seq**, RNA sequencing

**RT‐qPCR**, real‐time quantitative PCR

**siRNA**, small interfering RNA

**TMA**, Tissue‐MicroArray

**TSS**, transcription start site

**WHO**, world health organization

**XPDX**, XenoSTART Patient‐Derived Xenograft

Adenoid cystic carcinoma (ACC) is a rare cancer that accounts for about 1% of head and neck cancers and 20% of salivary gland malignancies in adults. It affects patients primarily in their fifth and sixth decades of life, but may appear at any age, with a slight predominance in women [1, 2]. ACC has a slow progression with 5‐year survival rates varying from 70 to 90%, but eventually most patients succumb to the disease [3]. The treatment options are limited due to ACC's inherent resistance to chemotherapeutic drugs and the so far limited response to tyrosine kinase inhibitors and immunotherapy [1, 4]. ACC typically grows in tubular, cribriform and/or solid patterns. The cribriform pattern is most prevalent and is characterised by nests of tumour cells with microcyst‐like spaces (ref WHO book). However, combinations of all three growth patterns are frequent. ACC tumour complexity is emphasised by its biphasic nature, with co‐proliferation of ductal and myoepithelial cells, the latter involved in paracrine signalling [5]. ACC's defining molecular characteristic is the t(6;9)(q22–23;p23–24) chromosomal translocation, leading to fusions of the *MYB* and *NFIB* transcription factor genes [4, 6, 7]. The prevalence of *MYB* rearrangements in head and neck ACC varies between 60% and 80% [8]. In addition to gene fusion, *MYB* overexpression by chromosome rearrangement can also be caused by super‐enhancers located within *NFIB*, *TGFBR3*, and *RAD51B* and its flanking sequences that are translocated in the proximity of the *MYB* promoter [9]. Notably, next‐generation sequencing has identified an overall low mutational burden, with pathogenic mutations in for example *PIK3CA*, *SMARCA2*, *CREBBP*, *NOTCH1*, *SPEN*, and *HRAS* [10, 11, 12].

The role of *MYB* as a driver of ACC is supported by studies showing upregulation of known direct targets of MYB, including genes involved in the apoptotic pathway, cell growth and angiogenesis, cell cycle control, DNA replication/repair, and cell adhesion [1, 11, 13]. Moreover, MYB inhibition by RNA interference or MYB‐targeted drugs decreases proliferation and spherogenesis of primary ACC cells and cause downregulation of known MYB target genes [14, 15, 16, 17]. However, there is no strong evidence that *MYB* alone can transform normal human epithelial cells. We previously observed increased proliferation rates of human MCF10A breast epithelial cells transduced with *MYB* or *MYB*‐*NFIB* fusion [13]. Treatment with a MYB inhibitor reduced the relative proliferation of these cells, indicating a *MYB*‐dependent effect. While *MYB*‐expressing epithelial cells acquired a partially transformed phenotype *in vitro*, they were unable to grow as xenografts in immunocompromised mice, suggesting that *MYB* is required but not sufficient for malignant transformation of glandular cells [13]. Rearrangement and overexpression of *MYBL1*, a gene closely related to *MYB*, has been detected in some ACC cases, bringing the percentage of ACCs with activation of either *MYB* or *MYBL1* to 93% [8, 18].

To identify actionable targets downstream of MYB in glandular cells, we introduced a conditional *MYB* construct into MCF10A breast epithelial cells, which led to the identification of the checkpoint kinase BUB1 as a direct MYB target gene. Importantly, *BUB1* expression was increased in ACC patient samples compared to normal salivary glands. The potential therapeutic value of BUB1 inhibition was studied in a cellular model and validated in MYB fusion‐positive ACC cells and organoids.

## Materials and methods

### Cell culture

MCF10A (obtained from the ATCC), a non‐tumorigenic human mammary epithelial cell line, was grown in DMEM/F12 medium (Gibco, Paisley, UK), 5% horse serum (v/v) (Gibco), and 100 U·mL^−1^ penicillin–streptomycin (PenStrep) antibiotic (Gibco), supplemented with 20 ng·mL^−1^ of human epidermal growth factor (hEGF) (Invitrogen, Inchinnan, UK), 500 ng·mL^−1^ hydrocortisone (MP Biomedicals, Graffenstaden, France), 100 ng·mL^−1^ cholera toxin (Invitrogen), 10 μg·mL^−1^ insulin (Sigma‐Aldrich, Gillingham, UK). HEK293T cells (ATCC) were cultured in DMEM GlutaMAX (4.5 mg·mL^−1^ glucose, L‐glutamine) (Gibco), supplemented with 10% v/v foetal bovine serum (FBS) (Gibco), and 100 U·mL^−1^ PenStrep antibiotic (Gibco). ACCX11 cells were propagated from a patient‐derived xenograft (PDX) [19]. Cells were monitored for mycoplasma using the MycoSensor Assay Kit (Agilent, Santa Clara, CA, USA).

### Proliferation assay

Cells were seeded in 96‐well black pigmented plates. After 72 h in culture with or without drugs, the alamarBlue Reagent (Thermo Fisher Scientific, Waltham, MA, USA) was added in an amount equal to 10% of the medium volume in the well. Plates were incubated for 4 h at 37 °C and 5% CO_2_. Next, plates were left for 15 min at room temperature before cell viability was measured by reading fluorescence at 544 nm.

### 3D drug screening assay with BUB1 inhibitor

Three‐dimensional (3D) ACC spheroids were derived from cryopreserved tissues extracted from ACCX11, ACCX6 and ACCX5M1 PDX models. Drug response was measured using the KIYA‐Predict™ assay [20, 21]. Briefly, tissues were enzymatically dissociated to single cells, evenly distributed within a 384‐well spheroid microplates (Corning, Glendale, CA, USA), and grown in KIYA‐Predict™ media (Kiyatec, Greenville, SA, USA) for 24 h prior to drug addition. Following 96 h of drug exposure, cell viability was measured using CellTiter‐Glo 3D (Promega, Madison, WI, USA). BUB1 inhibitor (BAY1816032, MedChemExpress, Monmouth Junction, NJ, USA) was used at a concentration range of 1–10 μm. Cell viability data were normalised to vehicle‐treated controls. Nonlinear regression curves and IC50s were calculated using graphpad prism (Boston, MA, USA).

### Lentiviral vectors and infections

The inducible lentiviral vector pINDUCER21 (ORF‐EG) backbone (hereby referred to as “EV”, empty vector, #46948) and pINDUCER21‐MYB (#51305) were purchased from Addgene (Watertown, MA, USA). The viral packaging vectors pCMV(‐PL) and pMD2.G (Addgen, #20783 and #12259, respectively) were used to assemble lentiviral particles after transfection of the lentivirus and packaging vectors in HEK293T using TurboFect Reagent (Invitrogen) mixed with Opti‐MEM (Gibco). Polybrene (Merk Millipore, Burlington, MA, USA) was added during transduction at a concentration of 5 μg·mL^−1^.

### Immunohistochemistry (IHC)

A XenoSTART PDX Tissue‐MicroArray (TMA) tissue section was stained with a rabbit monoclonal anti‐Bub1 antibody (clone EPR18947, 1 : 100 dilution; incubation overnight; cat. number ab195268; Abcam). Antigen retrieval was performed by microwave treatment at 750 W (10 min) in EDTA buffer (pH 8.0). A biotinylated goat anti‐rabbit horseradish peroxidase (HRP) secondary antibody (ab97200; Abcam) was used for signal amplification. DAB (3,3′‐Diaminobenzidine) was used as chromogen. Additional methods and references are included in supplementary Data S1.

## Results

### Establishing a new cellular model based on mammary epithelial cells containing a switchable *MYB* transgene

**Fig. 1:**
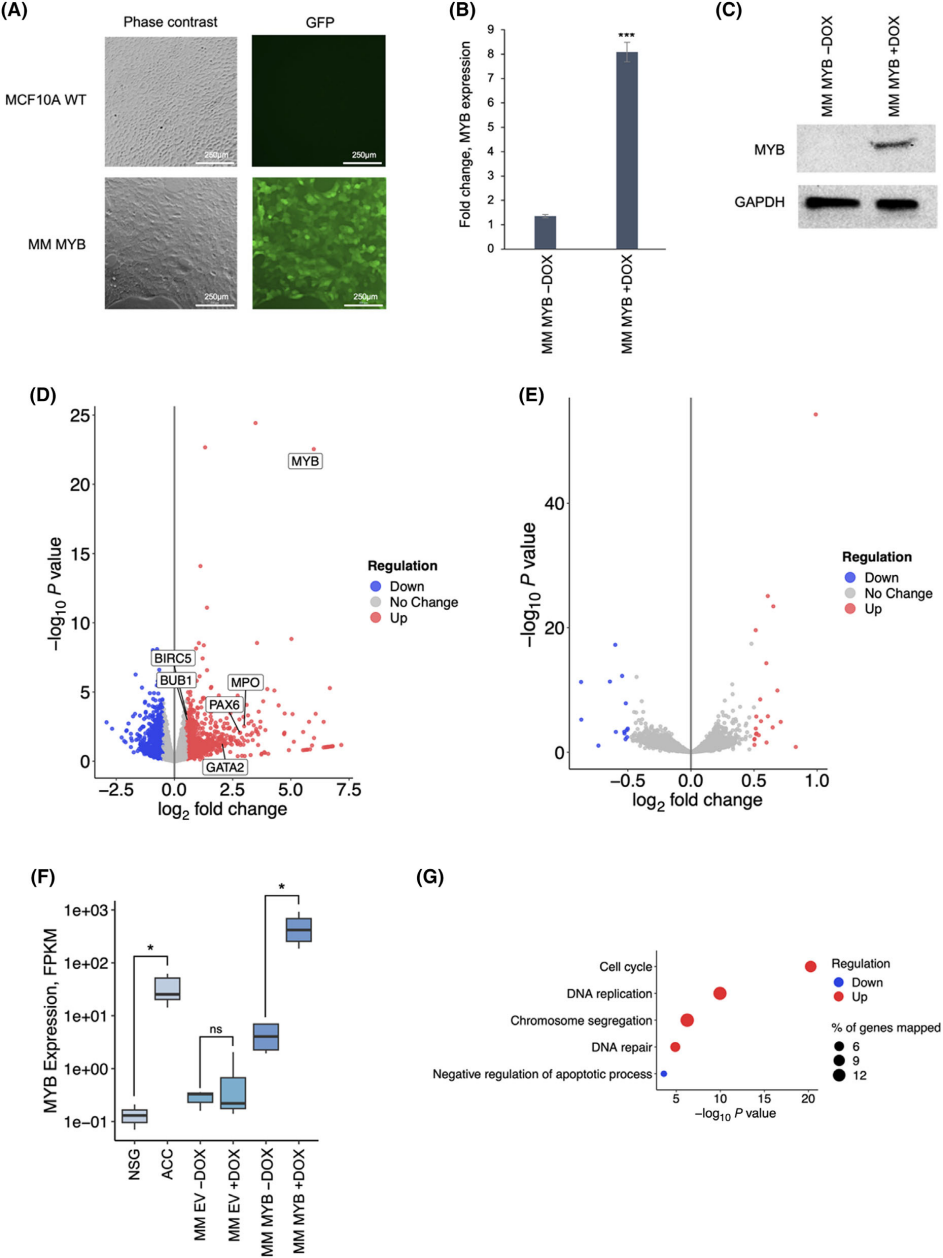
Generation of a new model of ACC based on MCF10A cells conditionally expressing *MYB*. (A) Fluorescence microscopy analysis of MCF10A cells transduced or non transduced with the lentiviral vector expressing MYB and GFP. Magnification used was 40×. (B) RT‐qPCR analysis showing *MYB* expression changes between −DOX and +DOX conditions. *MYB* expression was normalised to that of *GAPDH*. (C) Immunoblotting analysis demonstrating MYB protein expression in MM MYB in the presence of the inducer DOX. GAPDH protein levels were used as loading control. (D) Volcano plot depicting differentially expressed genes identified in the RNA‐seq analysis of MM MYB cells. Genes significantly up‐ or down‐regulated in MM MYB cells in the presence of DOX are indicated by the red or blue colour, respectively; known targets of MYB are highlighted. (E) Volcano plot depicting differentially expressed genes identified in the RNA‐seq analysis of MM EV cells. No significant changes in global gene expression are detected in control vector‐infected cells in the presence of DOX. (F) RNA‐seq analysis to detect expression of *MYB* in ACC cells (ACCX11, *n* = 7), NSG control (*n* = 3), MM EV (*n* = 3), and MM MYB (*n* = 3) cells cultured in the presence or absence of DOX. Expression is reported as fragments per kilo base of transcript per million mapped fragments (FPKM). (G) Bubble plot showing the GO terms of biological processes activated in MM cells after MYB activation, according to Panther:BP database. Results were filtered by *P* value ≤ 0.05 and adjusted by FDR ≤ 0.1. Different colours are used to distinguish biological processes. The size of the dots indicates the % of genes mapped for each biological process. Statistically significance was calculated using *t*‐test; *P* value ≤ 0.05 (*), 0.001 (**), 0.0001 (***), and 0.00001 (****); ns, not significant.

MCF10A cells were transduced with a lentiviral vector (pINDUCER21‐MYB) that drives expression of the human *MYB* gene when adding doxycycline (DOX) in the culture medium [22]. As a negative control, we used the empty vector (pINDUCER21‐EV). Successful vector transduction was verified by detecting expression of GFP by fluorescence microscopy (Fig. 1A). We established by flow cytometry analysis that the transduction efficiency was between 82% and 90% in the populations analysed (Fig. S1). To determine whether pINDUCER21‐MYB transduced MCF10A cells (called MM MYB cells) were able to express *MYB* in the presence of DOX, qPCR analysis was performed on MM MYB cells before and after addition of the inducer. *MYB* mRNA expression levels increased significantly in cells exposed to DOX (Fig. 1B). Moreover, immunoblotting confirmed the presence of MYB protein in MM MYB cells exposed to DOX, but not in untreated cells (Fig. 1C). RNA sequencing (RNA‐seq) analysis was performed on MM MYB cells with or without DOX treatment to identify genes differentially expressed after switching *MYB* on or off. A total of 798 differentially expressed genes were identified, of which 335 were upregulated and 463 downregulated in the presence of the inducer (Fig. 1D and Fig. S2A; the top 150 upregulated genes are reported in Table S1). Known *MYB* target genes, such as *GATA2*, *MPO*, *PAX6*, *BIRC5*, *BIRC1* were upregulated in the presence of DOX, validating the robustness of the system. To confirm specificity, the same analysis was performed on empty vector‐infected cells (MM EV) prior and after addition of DOX. RNA‐seq highlighted only minor changes in gene expression in these cells, confirming that the significant changes in global gene expression observed in DOX‐treated MM MYB cells was caused by *MYB* overexpression and not by the presence of DOX (Fig. 1E and Fig. S2B). Notably, the difference in *MYB* expression levels between patient‐derived ACCX11 cells and normal salivary glands was comparable to that between MM MYB in uninduced (−DOX) and induced (+DOX) conditions, although basal *MYB* levels were somewhat high in MM MYB cells even without DOX, probably due to promoter leakage (Fig. 1F). Biological processes associated with the differentially expressed genes in DOX‐treated MM MYB cells were highlighted by GO analysis. Cell cycle was confirmed as the top activated pathway, followed by DNA replication, chromosome segregation, and DNA repair (Fig. 1G).

**Fig. 2:**
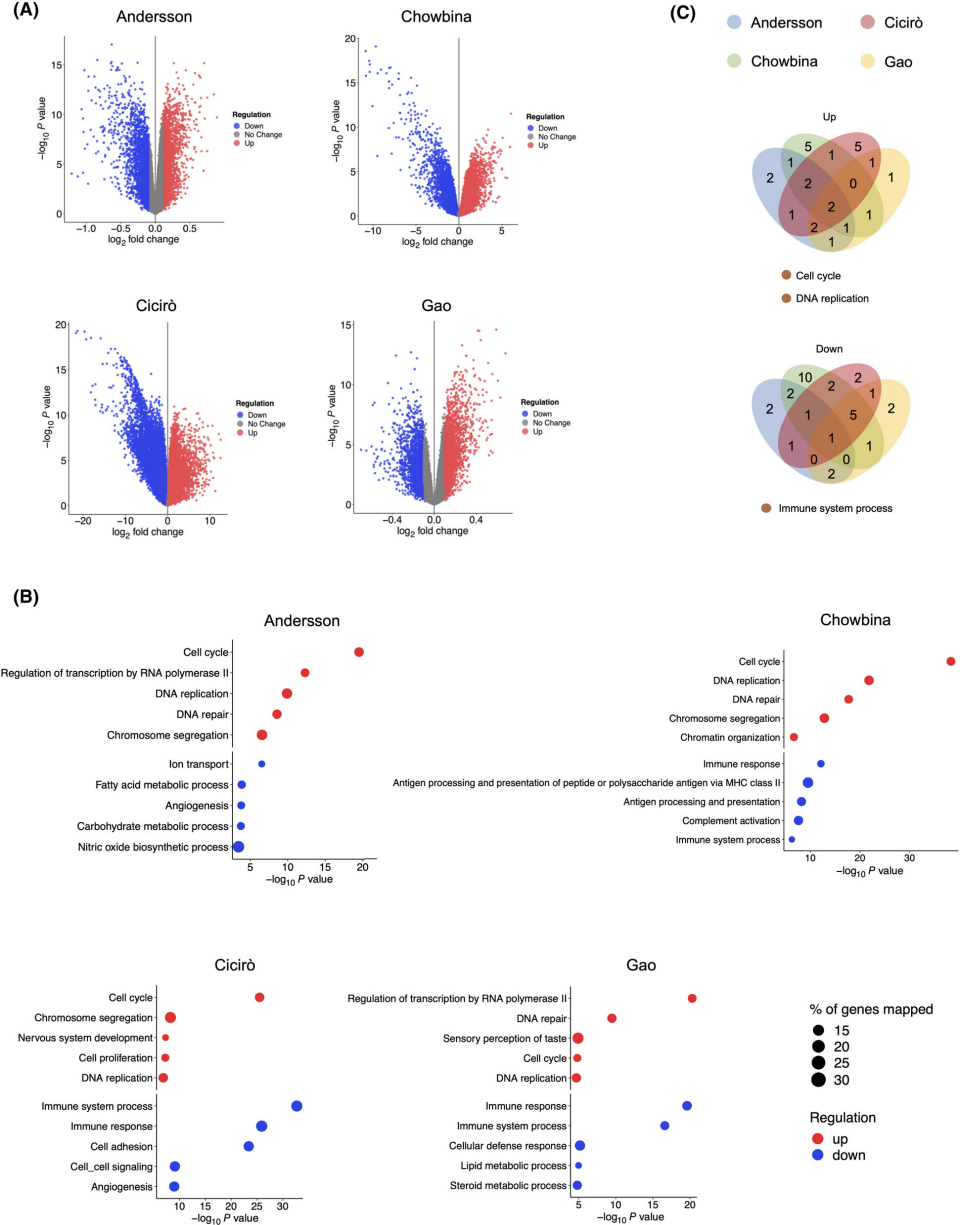
Genes differentially expressed in ACC versus NSG in publicly available datasets. (A) Volcano plots visualising differentially expressed genes (upregulated in red, and downregulated genes in blue) in the Andersson, Chowbina, Cicirò, and Gao datasets. (B) Bubble plot showing the GO terms of the top 5 biological processes representative of the up‐ (in red) or down‐(in blue)regulated genes in ACC versus NSG. Results were filtered by *P* value ≤ 0.05 and adjusted by FDR ≤ 0.1. The size of the dots indicates the % of genes mapped for each biological process. (C) Venn diagrams showing shared processes in the Andersson, Chowbina, Cicirò, and Gao datasets. “Up” indicates the differentially upregulated genes of each dataset, “Down” the differentially downregulated genes.

To understand if induction of *MYB* in MM MYB cells recapitulates gene expression patterns in primary ACCs and cultured MYB‐NFIB positive ACC cells, a gene expression signature for ACC was constructed and compared with the RNA‐seq analysis of *MYB*‐overexpressing MM MYB cells. To establish an ACC gene expression signature, we carried out RNA‐seq analysis of ACC‐X11 cells. Gene expression data from these cells (named Cicirò dataset), was combined with gene expression signatures downloaded from publicly available ACC microarray datasets (Andersson, Chowbina, and Gao data sets; details are in Table S2). Firstly, we identified genes differentially expressed in ACC tumour samples compared to normal salivary glands (NSG). Volcano plots were generated for each dataset and the number of up‐ or downregulated genes classified by statistical significance (Fig. 2A; the top 150 upregulated genes of each dataset are reported in Tables S3–S6).

To confirm the biological relevance of up‐ and downregulated genes in ACC for each dataset, GO analysis was performed using the Panther:BP database. Interestingly, the top scoring pathways were related to the cell cycle as well as regulation of chromatin and chromosomes (Fig. 2B). A list of the significantly activated pathways for each dataset is reported in Fig. S3. Next, the pathways extracted from each GO analysis were intersected, showing that cell cycle and DNA replication where shared processes, whereas the only shared process related to downregulated genes was the immune response (Fig. 2C).

**Fig. 3:**
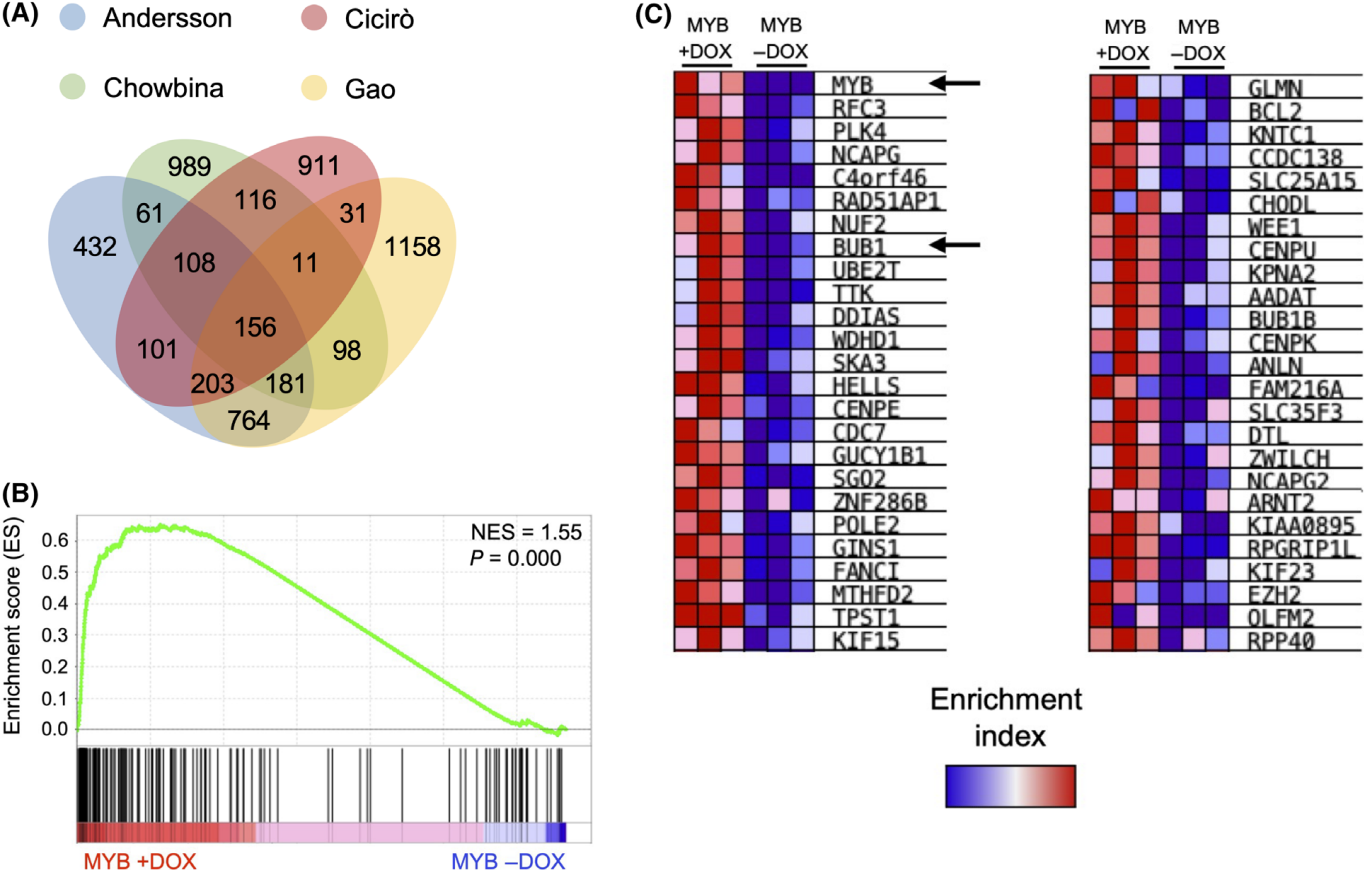
Generation of an ACC signature. (A) Venn diagram showing the intersection of the upregulated genes extrapolated from Andersson, Chowbina, Cicirò, and Gao datasets. (B) GSEA enrichment analysis of the 156 gene signature against RNA sequencing of MM MYB with (+) or without (−) DOX. (C) The top 50 core enriched genes (leading edge) extracted from GSEA recapitulated in the MM MYB + DOX model. The black arrows indicate *MYB* and *BUB1* genes.

To identify an ACC signature, significantly upregulated genes were intersected and plotted in a Venn diagram. The 156 upregulated genes shared between the four datasets were considered as a genuine ACC signature (Fig. 3A; the identity of each gene is listed in the Table S7). We performed Gene Set Enrichment Analysis (GSEA) to determine whether the 156 gene signature showed statistically significant enrichment in MCF10A cells with or without *MYB* overexpression. Analysis of the gene signature revealed a significant enrichment in MM MYB +DOX, but not in the −DOX condition, with a Normalised Enrichment Score NES of 1.55 and a *P* value of 10E‐16 (Fig. 3B). The top 50 core enriched genes (also known as ‘leading edge’) extracted from GSEA identified ACC‐specific genes recapitulated in the MM MYB +DOX model, which included the mitotic kinase BUB1 (Fig. 3C). This finding is supported by our previous observation of *BUB1* overexpression in MCF10A cells transduced with a retroviral vector driving expression of human *MYB* or *MYB‐NFIB* [13].

### Identification of the mitotic checkpoint kinase BUB1 as a MYB downstream target gene

**Fig. 4:**
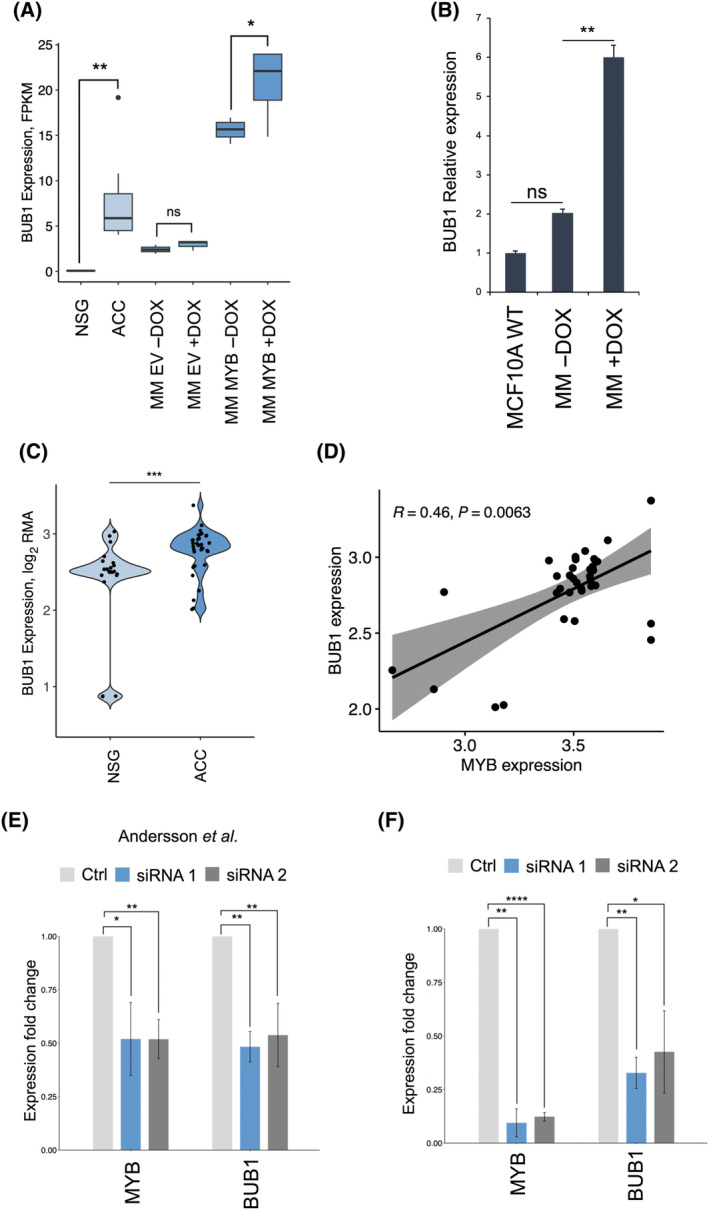
*BUB1* is a downstream target of *MYB*. (A) Boxplot quantifying *BUB1* expression in ACCX11 cells (*n* = 7), NSG (*n* = 3) and MM MYB (*n* = 3) and MM EV (*n* = 3) with or without DOX. (B) *BUB1* expression was quantified by RT‐qPCR in naïve MCF10A cells or MM MYB cells in the presence or absence of DOX. (C) Violin plot showing *BUB1* expression in the combined Andersson, Chowbina, Cicirò, and Gao datasets compared to NSG controls. Expression is quantified using log_2_ RMA (Robust Multi‐Array Average). (D) *MYB* and *BUB1* expression was extrapolated from publicly available datasets (Andersson, Chowbina, Cicirò, and Gao) and the statistical relationship between the two variables was calculated. The grey area represents the correlation confidence interval (CI). R indicates the Pearson correlation coefficient. (E) Bar plot showing the expression fold changes measured by microarray of *MYB* and *BUB1* genes in ACC cells treated with two independent siRNAs targeting *MYB‐NFIB*. The data was extrapolated from Andersson *et al*. [14]. (F) Expression levels of *MYB* and *BUB1* in ACCX11 cells exposed to MYB siRNAs. Bars indicate mean values plus or minus standard errors. Statistical significance was calculated using *t*‐test; *P* value ≤ 0.05 (*), 0.001 (**) and 0.00001 (****).

RNA‐seq analysis confirmed that genes related to the cell cycle and cell division were activated as a consequence of MYB overexpression. Among these genes, *BUB1* was upregulated in MM MYB cells upon addition of DOX and in ACCX11 cells compared to normal salivary glands (Fig. 4A). The result was further validated by qPCR (Fig. 4B). BUB1 is a mitotic checkpoint kinase whose gene expression has been linked to abnormal cell proliferation in salivary gland malignancies [23]. We therefore investigated if aberrant activation of *MYB* in ACC could lead to increased expression of the kinase, promoting cancer proliferation and/or drug resistance.

*MYB* and *BUB1* expression levels in the combined datasets were increased in primary ACC samples compared to NSG controls (Fig. 4C). Moreover, Pearson correlation showed a significant relationship between the expression levels of *MYB* and *BUB1* in patient tumours (Fig. 4D). To assess whether *BUB1* expression depends on *MYB* levels, we analysed a published microarray gene expression dataset in which *MYB‐NFIB* was downregulated by RNA interference in ACC cells [14]. Treatment with two independent siRNAs targeting *MYB‐NFIB* resulted in the downregulation of *MYB*, together with a concomitant decrease in *BUB1* expression compared to the scramble siRNA control (Fig. 4E). These results were validated using the same set of siRNAs in ACCX11 cells. RT‐qPCR analysis demonstrated that *BUB1* expression significantly declined after siRNA‐mediated knockdown of *MYB‐NFIB* (Fig. 4F).

### MYB transactivates the *BUB1* promoter and binds to the *BUB1* gene *in vivo*

**Fig. 5:**
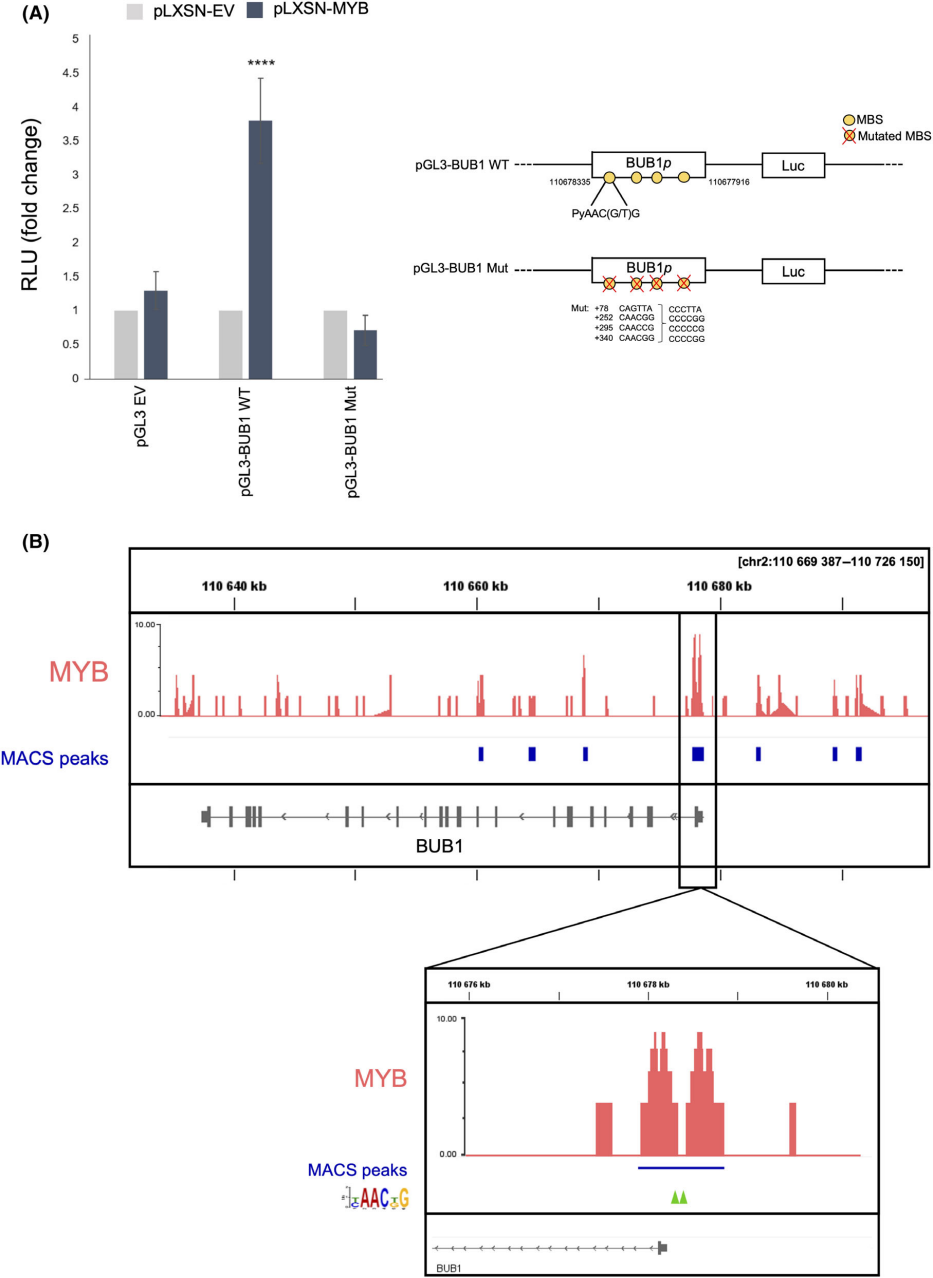
MYB transactivates the *BUB1* promoter via canonical MYB binding sequences. (A) Luciferase assay. The bar plot indicates the RLU (Relative Light Unit) values corrected for transfection efficiency with Renilla luciferase. pGL3 empty vector (pGL3 EV) was used to calculate background luciferase activity. A schematic representation of the constructs, the putative consensus MBSs (G/TAACNG) and the mutations introduced are shown on the right of the panel. Error bars indicate standard deviations. (B) Localisation of MYB peaks (depicted in red) detected by ChIP‐seq. The blue boxes under each peak indicate regions significantly enriched by the MYB binding as defined by the peak caller output of *P* < 0.01, calculated with the Model‐based Analysis of ChIP‐Seq (MACS) algorithm. MYB‐binding peaks around the transcription start site (TSS) of *BUB1* are highlighted in the zoomed box. The green arrows indicate the position of MYB canonical binding sites.

We hypothesised that MYB binds to and transcriptionally activate the *BUB1* gene. Manual inspection of the *BUB1* 5′ flanking region revealed several iterations of the G/TAACNG *MYB* consensus sequence in both DNA strands. Furthermore, using Cistrome DB online tools, we confirmed that there were chromatin immunoprecipitation (ChIP) MYB peaks overlapping with the putative MYB binding sequences in Jurkat leukaemia cells, which expresses high levels of *MYB* (not shown). To assess whether MYB could directly transactivate *BUB1* in glandular cells, MCF10A cells were co‐transfected with the wild‐type *BUB1* promoter region cloned in the pGL3 firefly basic vector (pGL3‐BUB1 WT), and a pLXSN vector expressing MYB (pLXSN‐MYB) [24]. There was a fourfold increase in luciferase activity in the presence of the pLXSN‐MYB vector, compared to the empty vector control (pLXSN‐EV), suggesting that the *BUB1* promoter was transactivated by MYB. To demonstrate that the effect was caused by direct interaction of MYB with the *BUB1* promoter, we used a *BUB1* promoter reporter plasmid (pGL3‐BUB1 Mut) in which all the canonical MYB binding sites (MBSs) were mutated (Fig. S4). As expected, mutation of the MYB‐binding sites completely abolished MYB transactivation, confirming specificity of the interaction (Fig. 5A).

To investigate whether endogenous MYB binds the *BUB1* gene in ACC cells, we examined genome‐wide MYB occupancy patterns in ACCX11 cells using chromatin immunoprecipitation sequencing (ChIP‐seq). We identified the presence of peaks in the *BUB1* promoter region, located at −0.84 Kb and −0.177 Kb from the transcription start site (TSS) of the gene, similarly to what was observed from data in Cistrome DB for leukaemia cells (Fig. 5B and data not shown). Interestingly, we found other peaks that overlapped putative enhancers in intragenic positions (Fig. 5B). Taken together, these results demonstrate that MYB directly binds to and transactivates the *BUB1* gene in glandular epithelial cells.

### Expression of *MYB* sensitises MCF10A cells to a BUB1 small molecule inhibitor

**Fig. 6:**
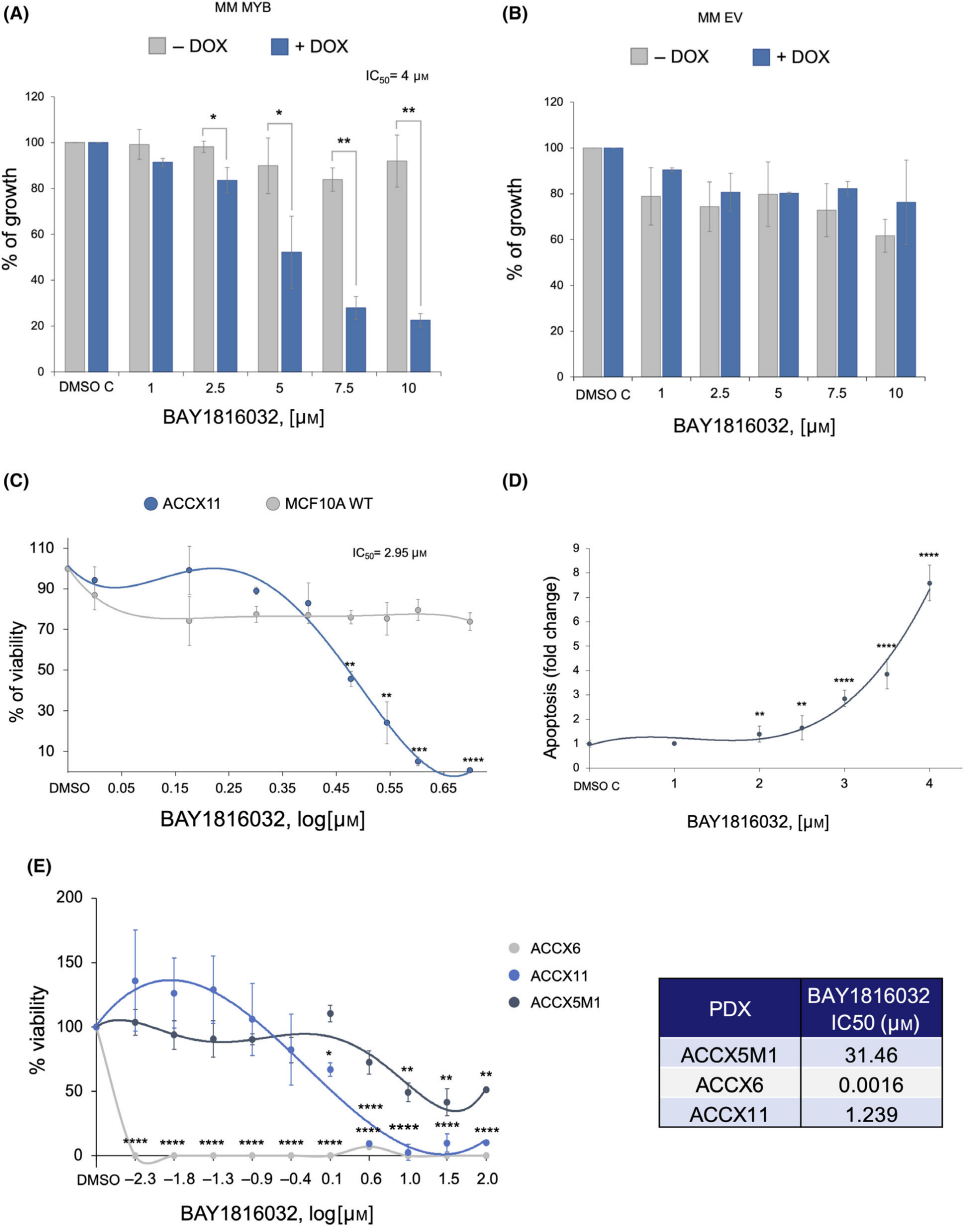
A BUB1 inhibitor impairs growth of glandular cells in *MYB*‐dependent manner and causes apoptosis of ACC patient‐derived cell lines. (A) Growth assay. MM MYB cells were treated with escalating doses of BAY1816032 (or vehicle DMSO) for 72 h in the presence or absence of the *MYB* inducer DOX. Growth of DMSO‐treated cells was considered 100%. (B) Growth assay was performed as in A except that the MM EV control cell line was used. (C) Viability assay. ACCX11 cells were exposed to escalating concentrations of BAY1816032 for 72 h. MCF10A were used as normal cell control. (D) Apoptosis assay. Caspase 3/7 activity was measured in ACCX11 cells treated for 48 h with BAY1816032. DMSO was used as treatment control. (E) Viability of ACCX6, ACCX11, and ACCX5M1 patient‐derived tumour spheroids cultured in 3D was measured after exposure to increasing concentrations of BAY1816032 for 96 h. The table indicates the IC_50_ obtained for each cell line. Error bars indicate standard errors. *P* value ≤ 0.05 (*), 0.001 (**), 0.0001 (***), and 0.00001 (****) (*n* = 6 technical replicates).

To evaluate the potential impact of BUB1 inhibition in *MYB*‐driven cancers, we treated MM MYB cells with BAY1816032, a selective inhibitor of the catalytic activity of the kinase [25]. In the presence of the inducer DOX, escalating concentrations of the drug caused loss of viability, with an IC_50_ of 4.0 μm, whereas in the absence of DOX‐induced *MYB* the drug had almost no effect (Fig. 6A). To rule out non‐specific interactions between DOX and the BUB1 inhibitor, we repeated the dose response experiment with MCF10A cells transduced with the empty, control virus (MM EV cells). As expected, no significant reduction in cell viability was detected in the presence of increasing concentrations of BAY1816032, with or without DOX treatment, demonstrating that the effect of the small molecule inhibitor is *MYB*‐dependent (Fig. 6B).

### The BUB1 inhibitor slows the growth and promotes apoptosis of patient‐derived ACC cells and spheroids

To verify if the BUB1 protein is expressed in ACC samples, we carried out immunohistochemical analysis on tissue microarray (TMA) sections, from a collection of ACC XenoSTART Patient‐Derived Xenograft (XPDX) models. Nine ACC samples were positive (82%), while two (18%) were negative for staining of BUB1. BUB1 protein was localised in the nucleus in five of the samples, whereas in the remaining samples three presented cytoplasmic and one diffuse staining (Fig. S5).

To validate BUB1 inhibition as a therapeutic strategy in ACC, we treated ACCX11 cells with BAY1816032 for 72 h. Pharmacological inhibition of BUB1 led to decreased cell viability in a dose‐dependent fashion with an IC_50_ of 2.95 μm. As expected, the treatment had no significant effect on naïve MCF10A cells, used as a *MYB*‐negative control cell line (Fig. 6C). To assess whether the kinase inhibitor was inducing tumour cell apoptosis, we performed a caspase activation assay. ACCX11 cells were treated with increasing concentrations of BAY1816032 for 48 h. A concentration of 2 μm of the drug was sufficient to produce a significant increase of caspase 3/7 activity, supporting the hypothesis that the BUB1 inhibitor induces programmed cell death in ACCX11 cells (Fig. 6D).

Changes in cell viability were also assessed in tumour spheroids. Three PDX tumours with high (ACCX11), medium (ACCX6), and low (ACCX5M1) BUB1 expression were selected for this analysis (Fig. S5). After 96 h of incubation with the BAY inhibitor, the viability of 3D spheroids was quantified. Consistent with the results obtained with 2D cultures, the viability of spheroids decreased as the drug concentration increased, with low IC50 values observed in cells expressing medium or high levels of BUB1 protein (Fig. 6E).

## Discussion

ACC is a rare malignancy characterised by a slow but relentlessly progressive course, often accompanied by resistance to therapy and ultimately death. Development of more effective targeted therapies can benefit from a better knowledge of the molecular and genetic alterations that are unique to this neoplasm. However, the lack of useful cellular ACC models has hindered the understanding of the biology of the disease. This study addresses three important aspects of ACC biology: (a) generation of an *in vitro* model to study the significance of *MYB*, (b) validation of *BUB1* as a target gene of MYB, (c) the potential use of BUB1 inhibition as a therapeutic strategy.

To address our first aim and investigate the significance of *MYB*, we engineered the human epithelial breast cell line MCF10A with a lentiviral vector carrying a *MYB* inducible cDNA under the control of doxycycline. We reasoned that the widely used immortalised, yet non‐tumourigenic, mammary gland epithelial cell line MCF10A could be useful to study the function of MYB in an epithelial cell background [13, 26]. The conditional system allowed us to perform functional experiments and provide new insights into the transcriptional activation of *MYB* in glandular cells. We performed a comprehensive comparison of differentially expressed genes between MCF10A cells with or without *MYB* overexpression in order to establish the biological relevance of the “MYB‐ON” system through GO analysis. Cell cycle, DNA replication, chromosome segregation, and DNA repair pathways were the top activated biological processes, suggesting an increased potential for proliferation in accordance with our previous observation in an MCF10A retroviral model engineered to stably overexpress *MYB* [13]. Interestingly, top activated pathways in +DOX condition were comparable with the ones observed in cultured *MYB*‐activated ACC cells. Furthermore, we compared these cells to microarray datasets available from public domain resources, revealing striking similarities in gene expression between MCF10A cells overexpressing *MYB* and ACC patient samples, strengthening the reliability of the engineered *in vitro* model for future works.

One of the genes consistently activated by *MYB* in ACC cellular models and patient samples is the checkpoint kinase gene *BUB1*, encoding a serine/threonine kinase required for attachment of microtubules to the kinetochore, essential for normal mitosis [27]. We showed that ectopic expression of MYB directly transactivates the *BUB1* gene by binding to its promoter region, in line with what was recently observed by Cheng and colleagues in prostate cancer [28]. Transactivation of *BUB1* by MYB may promote tumour development, since overexpression of BUB1 cause hyper‐activation of Aurora B activity, aneuploidy, and spontaneous and *MYC*‐induced transformation in a transgenic mouse model [29]. An orally available inhibitor of the BUB1 catalytic activity, BAY 1816032, was recently developed and shown to have anticancer activity against triple‐negative mammary cancer when combined with taxanes, PARP, and ATR inhibitors [25]. *MYB* overexpression sensitised MCF10A cells to treatment with BAY1816032 (Fig. 6) suggesting that BUB1 could be used as a predictive biomarker to guide therapy. To establish the importance of BUB1‐inhibition, we treated ACC cells or ACC organoids with the BUB1‐inhibitor. The drug had a profound impact on the proliferation and survival of ACC cells, highlighting its clinical potential in glandular cancers. Although sensitivity of ACC tumour cultures and spheres was broadly proportional to BUB1 expression levels, it should also be noted that the expression of other genes with an impact on BUB1, such as proapoptotic TP53, might be important in establishing sensitivity or resistance to the drug [30]. Because of the favourable pharmacokinetic profile and tolerability of BAY1816032, it may be combined with other drugs in future *in vitro* and *in vivo* investigations. One of the challenges associated with the strenuous efforts to develop ACC models is the scarcity of ACC patients and patient‐derived tumour material. To further complicate the generation of stable ACC cell lines, cultured ACC cells lose histological and genomic characteristics after prolonged passages *in vitro* [31]. Studies of ACC PDXs have reported reassuring results in cellular maintenance, however, *in vivo* growth of tumour cells is time‐consuming, expensive, and the tumour engraftment capacity is not always fulfilled [32]. Our *MYB* conditional model, based on MCF10A cells, with all its limitations, could therefore be a useful cut‐price alternative to more complex cellular and PDX models for studies of MYB functions and drug screening.

## Author contributions

YC: study design, data acquisition, data analysis and interpretation, statistical analysis, manuscript preparation. DR: Data acquisition, data analysis and interpretation, statistical analysis. PTN: Data acquisition. RL and GS: data acquisition, data analysis. TDR and MM: data acquisition and analysis. MKA and GS: data analysis, manuscript editing. AS: study concepts, study design, manuscript preparation, funding.

### Peer review

The peer review history for this article is available at https://www.webofscience.com/api/gateway/wos/peer‐review/10.1002/1873‐3468.14786.

## Supporting information


**Fig. S1.** Flow cytometry strategy used for assessment of lentivirus transduction efficiency.
**Fig. S2.** RNA‐seq analysis of MM MYB and MM EV cells in the presence or absence of doxycycline (DOX).
**Fig. S3.** GO analysis of ACC and MM MYB datasets.
**Fig. S4.** BUB1 promoter segments cloned into the pGL3‐Basic luciferase vector.
**Fig. S5.** BUB1 protein expression in ACC PDX tissue microarrays.

**Table S1.** Top 150 genes upregulated in MM MYB +DOX, compared to MM MYB −DOX. P ADJ: p values adjusted by false discovery rate (FDR). Genes were ordered by decreasing log2 fold change.
**Table S2.** Details of the datasets.
**Table S3.** Top 100 upregulated genes in ACC cells compared to normal salivary glands in the Andersson database.
**Table S4.** Top 150 upregulated genes in ACC cells compared to normal salivary glands in the Chowbina database.
**Table S5.** Top 150 upregulated genes in ACC cells compared to normal salivary glands in the Chowbina database.
**Table S6.** Top 100 upregulated genes in ACC cells compared to normal salivary glands in the Gao database.
**Table S7.** ACC gene patient signature.

**Data S1.** Supplementary methods and references.

## Data Availability

ChIP‐seq data on ACCX11 cells are available at ArrayExpress accession E‐MTAB‐12976. Microarray datasets were downloaded from the Gene Expression Omnibus (GEO, https://www.ncbi.nlm.nih.gov/geo/) repository. The Andersson dataset (GEO accession number GSE88804) reported expression data of 13 surgical samples of ACC and 7 NSG samples [8]. The Chowbina dataset (GEO accession number GSE36820) presented microarray analysis on 3 normal samples and 11 ACC xenograft samples. The Gao dataset (GEO accession number GSE59702) contains expression profiling by array of 12 ACC with matched normal tissues [9]. RNAseq data from Cicirò dataset report 7 replicates of ACC samples (ACCX11) and 3 NSG samples; data available at ArrayExpress accession E‐MTAB‐12978. RNAseq data of MM MYB/EV ± DOX are available at ArrayExpress accession E‐MTAB‐12977.
