# Supplementary material for: The mitotic checkpoint kinase BUB1 is a direct and actionable target of MYB in adenoid cystic carcinoma
Source: FEBS Lett. 2023 Dec 27;598(2):252–65. doi: 10.1002/1873-3468.14786 (PMC11774229; doi:10.1002/1873-3468.14786)
Supplement: Supplementary file 1 — Fig. S1. Flow cytometry strategy used for assessment of lentivirus transduction efficiency. Fig. S2. RNA‐seq analysis of MM MYB and MM EV cells in the presence or absence of doxycycline (DOX). Fig. S3. GO analysis of ACC and MM MYB datasets. Fig. S4. BUB1 promoter segments cloned into the pGL3‐Basic luciferase vector. Fig. S5. BUB1 protein expression in ACC PDX tissue microarrays. [file FEB2-598-252-s001.docx]

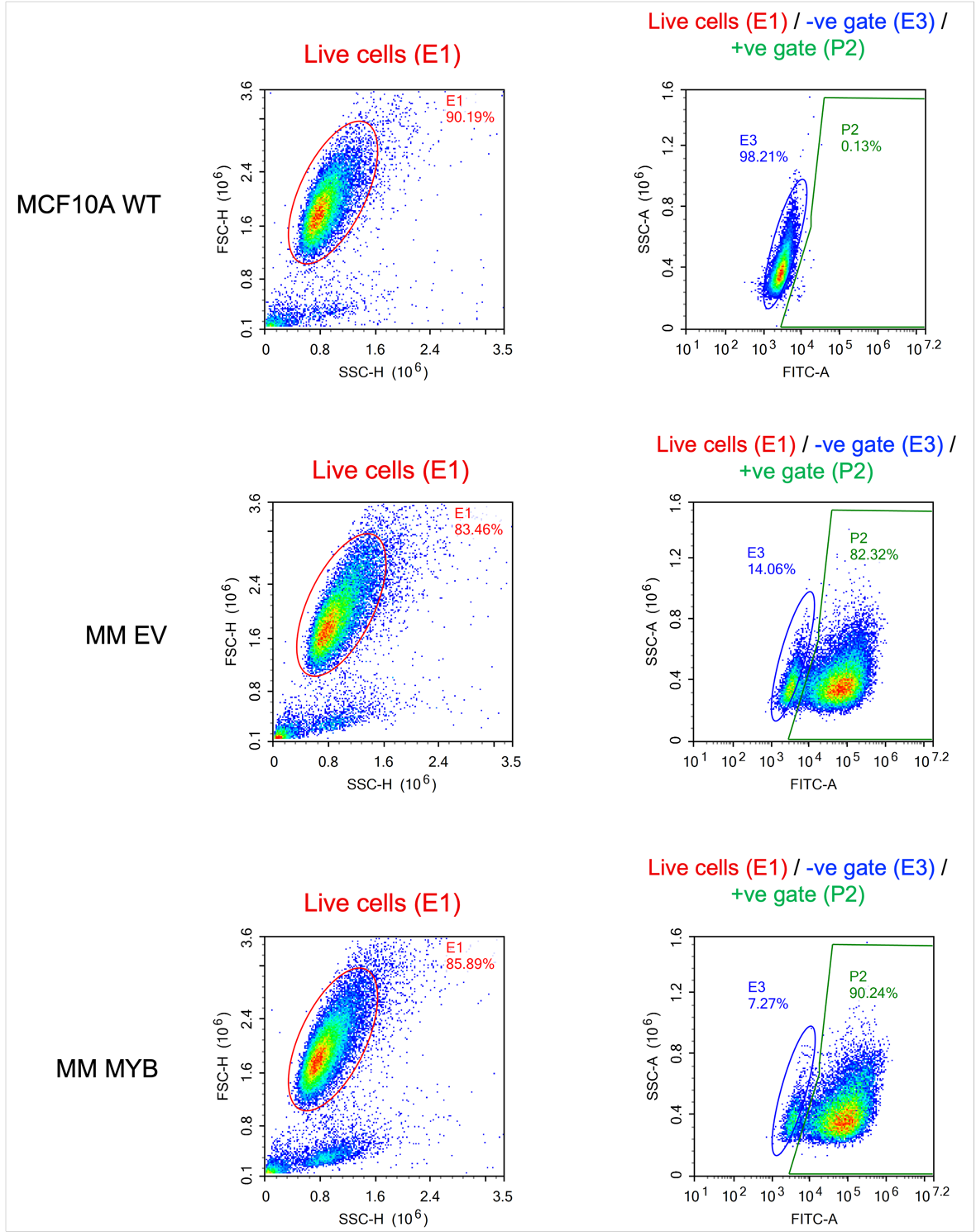


**Supplementary Figure 1. Flow cytometry strategy used for assessment of lentivirus transduction efficiency.** Live MCF10A parental cells (WT) were used as non-transduced control (top panels). The same gating was applied for MCF10A cells transduced with pINDUCER21-EV vector (MM EV, middle panels) or pINDUCER21-MYB vector (MM MYB, bottom panels). Percentages of GFP positive cells is showed in gated P2 areas.


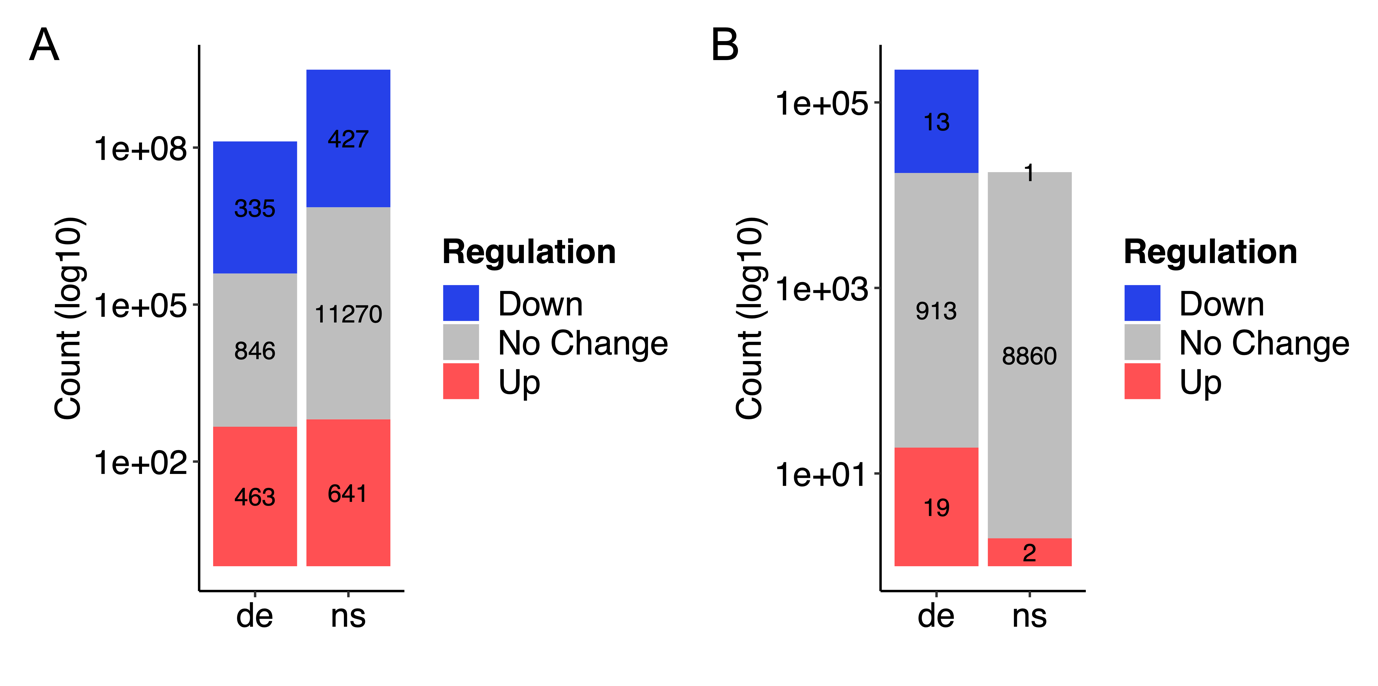


**Supplementary Figure 2. RNA-seq analysis of MM MYB and MM EV cells in the presence or absence of doxycycline (DOX). A)** Number of genes up- (red) or downregulated (blue) in MM MYB cells or in **B)** MM EV cells. A total of 798 differentially expressed genes were found in MM MYB +DOX *vs* -DOX condition and divided as up- or downregulated according to their fold change, with a fold change threshold ≥ ± 1 and an FDR cut-off of 0.05.


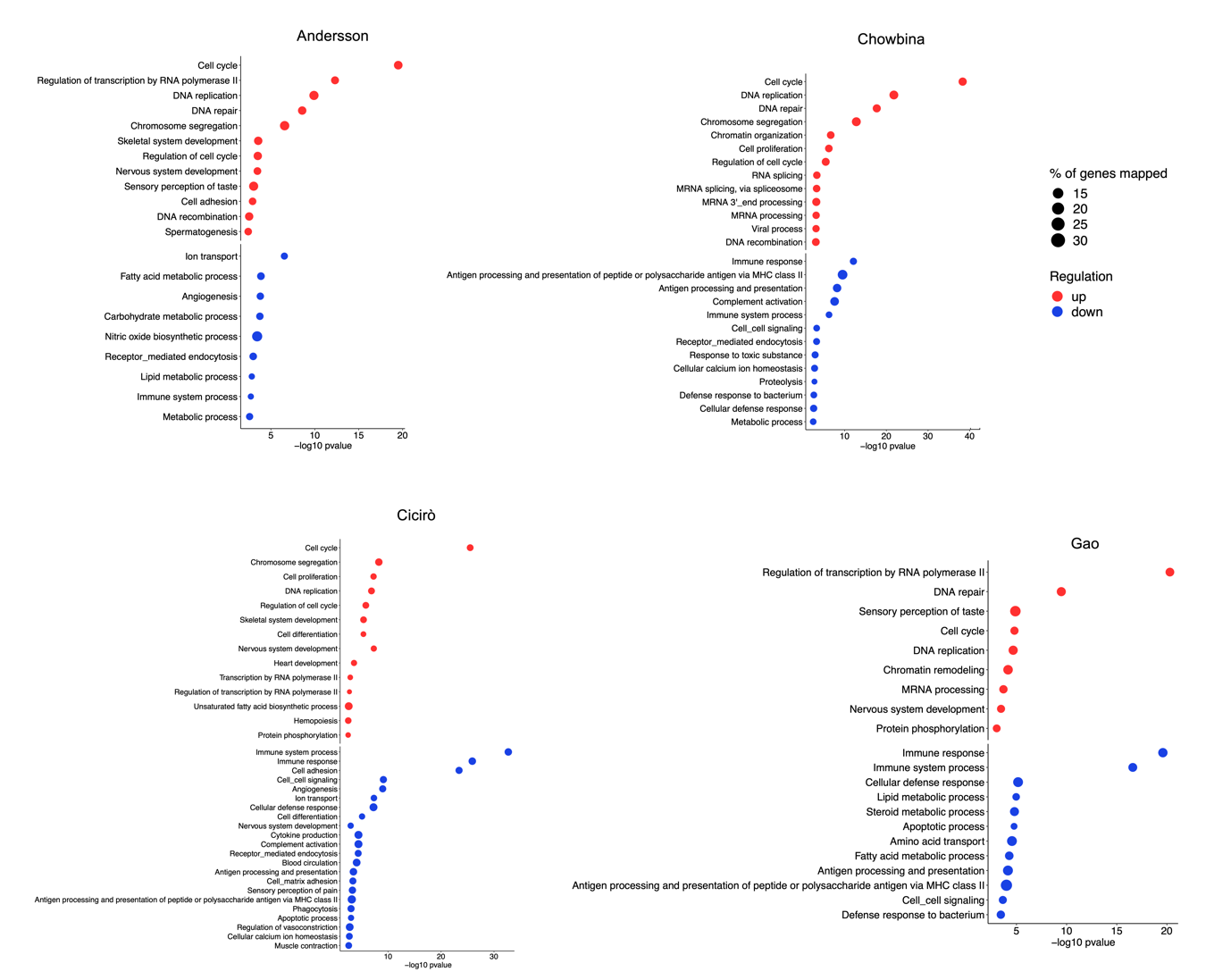


**Supplementary Figure 3. GO analysis of ACC and MM MYB datasets.** Dot plot showing the biological processes most significantly up- or downregulated in the Andersson, Chownina, Cicirò, and Gao dataset, selected with a p value ≤ 0.05 and filtered by FDR ≤ 0.1. The size of the dots indicates the percentage of the genes mapped in each biological process, and up- and downregulated genes are shown in blue and red, respectively.


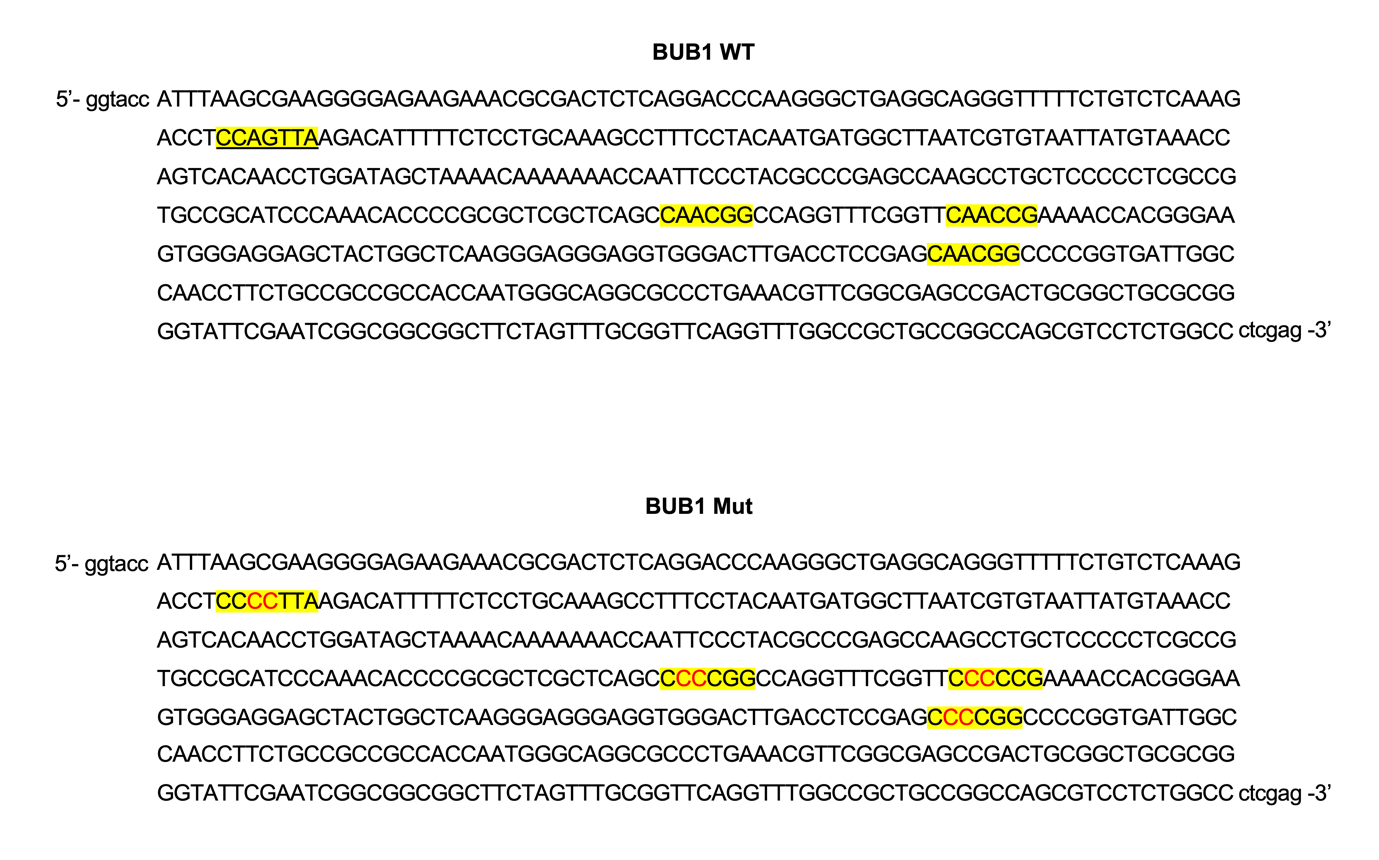


**Supplementary figure 4. BUB1 promoter segments cloned into the pGL3-Basic luciferase vector.** A 500 bp upstream of the transcriptional start site were cloned into the pGL3-basic luciferase vector in the wild type conformation (upper panel) or containing mutations in the putative MYB binding sites (MYB binding sites and mutations highlighted in yellow). The MYB binding site located in the lower strand is underlined. In lowercase the sequence of the restriction enzymes used to create sticky ends to facilitate cloning.


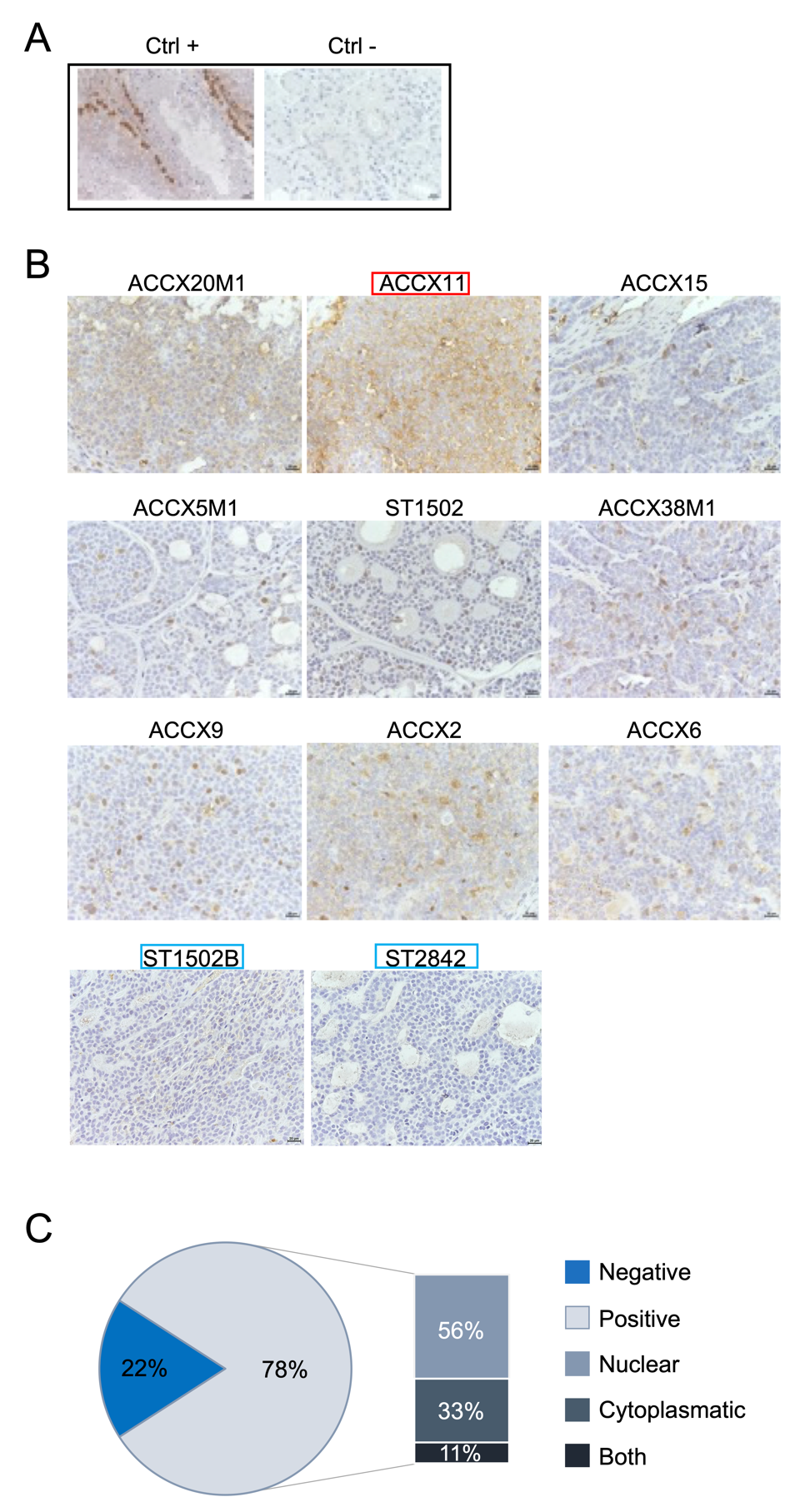


**Supplementary Figure 5.** **BUB1 protein expression in ACC PDX tissue microarrays. A)** Immunohistochemical analysis showing expression of BUB1 in mouse testis (positive control, Ctr +) and normal parotid gland (negative control Ctrl -). **B)** Sections from eleven independent ACC PDXs were stained with a BUB1 specific antibody. The red box highlights ACCX11 cells, the blue boxes negative salivary tumour tissues. **C)** Schematic representation of the ACC samples classified according to the localisation of BUB1 protein, expressed in percentages.
