# Supplementary material for: The mitotic checkpoint kinase BUB1 is a direct and actionable target of MYB in adenoid cystic carcinoma
Source: FEBS Lett. 2023 Dec 27;598(2):252–65. doi: 10.1002/1873-3468.14786 (PMC11774229; doi:10.1002/1873-3468.14786)
Supplement: Supplementary file 2 — Table S1. Top 150 genes upregulated in MM MYB +DOX, compared to MM MYB −DOX. P ADJ: p values adjusted by false discovery rate (FDR). Genes were ordered by decreasing log2 fold change. Table S2. Details of the datasets. Table S3. Top 100 upregulated genes in ACC cells compared to normal salivary glands in the Andersson database. Table S4. Top 150 upregulated genes in ACC cells compared to normal salivary glands in the Chowbina database. Table S5. Top 150 upregulated genes in ACC cells compared to normal salivary glands in the Chowbina database. Table S6. Top 100 upregulated genes in ACC cells compared to normal salivary glands in the Gao database. Table S7. ACC gene patient signature. [file FEB2-598-252-s003.docx]

**Supplementary Table 1.** Top 150 genes upregulated in MM MYB +DOX, compared to MM MYB -DOX. P ADJ: p values adjusted by false discovery rate (FDR). Genes were ordered by decreasing log2 fold change.

| **GENE** | **LOG2 FOLD CHANGE** | | **P VALUE** | **P ADJ** | **CONFIDENCE** |
| --- | --- | --- | --- | --- | --- |
| CTAGE11P | 6.67564285 | | 5.22E-06 | 0.00280043 | **** |
| ZNF853 | 6.41044345 | | 0.00143252 | 0.09812999 | *** |
| CCDC106 | 6.06721555 | | 0.00037739 | 0.05092857 | *** |
| TRIM71 | 5.99605089 | | 0.02080336 | 0.3718237 | * |
| **MYB** | **5.98653518** | | **2.86E-23** | **1.19E-19** | ******** |
| SLC47A1 | 5.78188068 | | 0.00141764 | 0.09788213 | *** |
| LINC02593 | 5.52974169 | | 0.0077191 | 0.23770867 | ** |
| ELOVL2 | 5.12948524 | | 0.03915181 | 0.48151228 | * |
| MX2 | 5.07396029 | | 0.00039308 | 0.05188669 | *** |
| CEMIP | 5.02594102 | | 1.48E-09 | 3.52E-06 | **** |
| KIF5C | 4.73539775 | | 0.00035654 | 0.0494397 | *** |
| HOXC10 | 4.72867763 | | 0.00868309 | 0.25128096 | ** |
| COLGALT2 | 4.71389632 | | 0.0109513 | 0.28197293 | ** |
| COL25A1 | 4.7089261 | | 0.0101701 | 0.27254699 | ** |
| A2M | 4.47493523 | | 9.21E-05 | 0.02129109 | **** |
| PLA2G4E | 4.29262642 | | 7.87E-06 | 0.00396987 | **** |
| RPP25 | 3.98636849 | | 6.14E-06 | 0.00319178 | **** |
| CXCL10 | 3.90672142 | | 0.00564191 | 0.20735399 | ** |
| UNC5C | 3.812519 | | 4.69E-05 | 0.01369383 | **** |
| HPCAL4 | 3.69355132 | | 0.00138456 | 0.09759187 | *** |
| MOXD1 | 3.68504082 | | 0.00354942 | 0.1640622 | ** |
| KLF17 | 3.61969008 | | 0.00026157 | 0.04225736 | *** |
| USP44 | 3.60301963 | | 0.01144022 | 0.28370386 | ** |
| ADAM22 | 3.56427792 | | 0.00780231 | 0.23865882 | ** |
| RNF150 | 3.54726488 | | 2.87E-09 | 5.40E-06 | **** |
| EYA2 | 3.51299177 | | 0.00465006 | 0.18873054 | ** |
| LAMA4 | 3.50090644 | | 0.00684436 | 0.22783916 | ** |
| GSDMA | 3.48388969 | | 3.79E-25 | 3.15E-21 | **** |
| RSPH10B2 | 3.40927884 | | 0.00253491 | 0.13433434 | ** |
| UAP1L1 | 3.36432557 | | 0.02943164 | 0.42660494 | * |
| PPP1R14A | 3.33939092 | | 0.00909281 | 0.25619199 | ** |
| PARVB | 3.30850402 | | 0.00130658 | 0.09509731 | *** |
| WNT11 | 3.27219843 | | 0.01266999 | 0.2965241 | ** |
| LHX6 | 3.18218397 | | 0.00186503 | 0.11439695 | *** |
| GBP4 | 3.15909797 | | 0.00048611 | 0.05844256 | *** |
| RARB | 3.15018049 | | 0.03517418 | 0.4630525 | * |
| ALPK2 | 3.11408046 | | 0.01253081 | 0.29409415 | ** |
| CDK15 | 3.10107992 | | 8.53E-05 | 0.02028812 | **** |
| DMC1 | 2.99419456 | | 0.03134051 | 0.44062613 | * |
| MPO | 2.99384431 | | 0.00295788 | 0.14825042 | ** |
| TMEM121 | 2.96295148 | | 0.00062834 | 0.06651479 | *** |
| ADAMTS17 | 2.90068922 | | 0.01972904 | 0.36427864 | ** |
| GAREM2 | 2.82324123 | | 0.00872236 | 0.25154256 | ** |
| ZYG11A | 2.81745818 | | 0.04739397 | 0.52020818 | * |
| PAX6 | 2.80629711 | | 0.00891856 | 0.2545537 | ** |
| WNT5B | 2.79019376 | | 0.0009519 | 0.08336672 | *** |
| ITGA7 | 2.73484172 | | 0.03389277 | 0.4543679 | * |
| CHST4 | 2.71445823 | | 1.82E-05 | 0.00740066 | **** |
| DERL3 | 2.70868822 | | 0.05788757 | 0.55445873 | * |
| PELI2 | 2.70090058 | | 0.05410151 | 0.54193107 | * |
| BHLHE41 | 2.68408902 | | 9.58E-05 | 0.02184709 | **** |
| ADAMTS3 | 2.64185675 | | 0.03604746 | 0.46679354 | * |
| NR2F1 | 2.63670643 | | 0.00432182 | 0.18280904 | ** |
| GFI1 | 2.62588098 | | 0.03392479 | 0.4543679 | * |
| LINC01085 | 2.55108655 | | 0.00652845 | 0.22570704 | ** |
| MESP1 | 2.55100208 | | 0.01859489 | 0.35978944 | ** |
| HOXA11-AS | 2.45537599 | | 0.02892552 | 0.42310609 | * |
| PPM1E | 2.44343073 | | 0.02071839 | 0.37104693 | * |
| SOWAHA | 2.44172499 | | 0.03053917 | 0.43550621 | * |
| SCARF2 | 2.40788868 | | 0.01662134 | 0.34077634 | ** |
| GPAT3 | 2.3992884 | | 0.00034961 | 0.04888691 | *** |
| HOXB6 | 2.39457105 | | 0.02738929 | 0.41339984 | * |
| LRCH2 | 2.39215334 | | 0.05244852 | 0.5390341 | * |
| HOXC4 | 2.38625159 | | 0.02141853 | 0.37648656 | * |
| APBB1 | 2.37268651 | | 0.00374052 | 0.16953228 | ** |
| IFFO1 | 2.35566254 | | 0.00425705 | 0.18138091 | ** |
| HOXA11 | 2.29748034 | | 0.00419663 | 0.17997937 | ** |
| JCAD | 2.28016635 | | 0.00189057 | 0.11439695 | *** |
| ARHGEF10 | 2.24961958 | | 0.01375803 | 0.31126139 | ** |
| NRIP3 | 2.24258 | | 0.04937758 | 0.52804819 | * |
| RFLNB | 2.2396897 | | 0.00077598 | 0.07426947 | *** |
| PI4KAP1 | 2.20199729 | | 0.02453598 | 0.3960027 | * |
| NCAM1 | 2.19635346 | | 0.05797826 | 0.55445873 | * |
| LARGE2 | 2.15328869 | | 0.04591017 | 0.51438136 | * |
| ADAM23 | 2.14893221 | | 0.00186702 | 0.11439695 | *** |
| SHISA2 | 2.14445261 | | 0.05295493 | 0.5390341 | * |
| NR6A1 | 2.13141662 | | 0.00436406 | 0.18374337 | ** |
| ADRA2C | 2.12805402 | | 0.03203678 | 0.44377329 | * |
| NRARP | 2.09302555 | | 0.00314976 | 0.15354264 | ** |
| CHDH | 2.0901246 | | 0.01166954 | 0.28640296 | ** |
| ARHGAP44 | 2.07987014 | | 0.05227344 | 0.5390341 | * |
| DNM1 | 2.06672226 | | 0.02859555 | 0.421526 | * |
| GATA2 | 2.04928313 | | 0.05046522 | 0.53452656 | * |
| CCDC74B | 2.03367135 | | 0.01967006 | 0.36367755 | ** |
| REEP2 | 2.03075309 | | 0.01107393 | 0.28197293 | ** |
| VSIG10L | 2.02299408 | | 0.01915365 | 0.36217804 | ** |
| PCOLCE2 | 1.97688882 | | 0.00115337 | 0.09095739 | *** |
| CAND2 | 1.97642504 | | 0.02634218 | 0.4051855 | * |
| JAM3 | 1.96121381 | | 0.01714464 | 0.34676723 | ** |
| CXCL11 | 1.93278443 | | 0.02439019 | 0.39546139 | * |
| CDHR3 | 1.92142508 | | 0.01910888 | 0.36217804 | ** |
| MPP2 | 1.91588535 | | 0.0340666 | 0.45458552 | * |
| DSCAM | | 1.9075525 | 1.78E-05 | 0.00740066 | **** |
| FLNC | | 1.90263943 | 0.02792998 | 0.41706196 | * |
| YTHDF3-DT | | 1.89334562 | 0.05793203 | 0.55445873 | * |
| NTN1 | | 1.87697236 | 0.00813805 | 0.242249 | ** |
| CCDC74A | | 1.81253868 | 0.00336514 | 0.15907917 | ** |
| FRMPD1 | | 1.80773137 | 0.02011514 | 0.36592939 | * |
| SLC16A9 | | 1.80650552 | 0.00735584 | 0.23496184 | ** |
| DPF1 | | 1.7992523 | 0.04057637 | 0.4889144 | * |
| TMEM74 | | 1.79738299 | 0.00296807 | 0.14831458 | ** |
| MYO1F | | 1.78372722 | 0.04707059 | 0.51871171 | * |
| COL6A1 | | 1.77040339 | 0.00163894 | 0.10694887 | *** |
| MANCR | | 1.74610462 | 0.00275807 | 0.14121323 | ** |
| DIP2C | | 1.73884169 | 0.0452277 | 0.50884987 | * |
| CTH | | 1.73477489 | 0.0105947 | 0.27589317 | ** |
| SIX1 | | 1.73181741 | 0.00680514 | 0.22783916 | ** |
| MX1 | | 1.71253344 | 0.04794082 | 0.52209277 | * |
| PRLR | | 1.68312951 | 0.01965938 | 0.36367755 | ** |
| UCHL1 | | 1.68018326 | 0.05924684 | 0.55813155 | * |
| ADCY1 | | 1.67566993 | 0.05308827 | 0.5390341 | * |
| ARHGEF25 | | 1.65211116 | 0.05221669 | 0.5390341 | * |
| LNP1 | | 1.6508822 | 0.04254225 | 0.49788036 | * |
| AUTS2 | | 1.65086574 | 0.01559888 | 0.33107819 | ** |
| DDIT4L | | 1.63889569 | 0.03282798 | 0.44701928 | * |
| MAP6D1 | | 1.63673005 | 0.05351076 | 0.54051968 | * |
| TTPA | | 1.6201812 | 0.03305848 | 0.44826913 | * |
| YBX2 | | 1.61408859 | 0.02399139 | 0.39342354 | * |
| HLX | | 1.61079715 | 0.03711296 | 0.47162151 | * |
| P3H3 | | 1.60970723 | 0.02964188 | 0.42738027 | * |
| RADX | | 1.59753334 | 0.00106132 | 0.08780586 | *** |
| TSNAXIP1 | | 1.58419989 | 4.32E-06 | 0.00256914 | **** |
| STMN3 | | 1.56391713 | 5.08E-06 | 0.00280043 | **** |
| COL18A1 | | 1.56360133 | 0.00030417 | 0.04510051 | *** |
| ADAM12 | | 1.55802449 | 0.00403935 | 0.17688083 | ** |
| CDH16 | | 1.55473166 | 0.00059197 | 0.063471 | *** |
| SCML2 | | 1.54891885 | 0.01120118 | 0.28217293 | ** |
| CMPK2 | | 1.54746745 | 0.00168273 | 0.1081108 | *** |
| IFI27 | | 1.51332161 | 0.01192952 | 0.28754423 | ** |
| PABPC4L | | 1.50005839 | 0.04045429 | 0.48815044 | * |
| CAPN5 | | 1.49941622 | 0.0260073 | 0.40449508 | * |
| SELPLG | | 1.47962574 | 0.03708527 | 0.47162151 | * |
| HIC2 | | 1.47761022 | 0.03169731 | 0.44211501 | * |
| GLDC | | 1.47084907 | 0.02345293 | 0.38947788 | * |
| SDC2 | | 1.4679401 | 0.02834314 | 0.4207225 | * |
| ARHGAP33 | | 1.46453715 | 0.01367154 | 0.31120987 | ** |
| LOC100996437 | | 1.45542209 | 0.04878582 | 0.52509449 | * |
| OAS2 | | 1.44468901 | 0.05534769 | 0.54560759 | * |
| ERFE | | 1.42985875 | 0.02171557 | 0.37782695 | * |
| CD302 | | 1.40285797 | 0.03986401 | 0.48584714 | * |
| PRDM8 | | 1.40140593 | 0.04628894 | 0.5147575 | * |
| MALT1-AS1 | | 1.39949794 | 2.66E-07 | 0.00024614 | **** |
| EEPD1 | | 1.39674061 | 0.01186046 | 0.28644118 | ** |
| CENPT | | 1.39628817 | 8.04E-12 | 2.23E-08 | **** |
| UCA1 | | 1.39598345 | 0.01056725 | 0.2756099 | ** |
| LOC100506071 | | 1.37750893 | 0.03749107 | 0.47333189 | * |
| ARHGEF6 | | 1.37612199 | 0.03723852 | 0.47265364 | * |
| COL13A1 | | 1.3617793 | 0.03952491 | 0.48466797 | * |
| CENPV | | 1.35356595 | 0.0008818 | 0.08092189 | *** |
| ADAMTS15 | 1.35140828 | | 0.01069441 | 0.27762081 | ** |

**Supplementary Table 2.** Details of the datasets.

| **DATASET** | **ACCESSION NUMBER** | **NUMBER OF ACC SAMPLES** | **NUMBER OF NSG SAMPLES** | **REFERENCE** | |
| --- | --- | --- | --- | --- | --- |
|  |  |  |  |  |  |
| Andersson | GSE88804 | 13 | 7 | | (Andersson *et al.*, 2017) |
|  |  |  |  |  |  |
| Chowbina | GSE36820 | 11 | 3 | | / |
|  |  |  |  |  |  |
| Cicirò | E-MTAB-12978 | 7 | 3 | | This study |
| Gao | GSE59702 | 12 | 12 | | (Gao, R. *et al*., 2014) |
|  |  |  |  |  |  |

**Supplementary Table 3.** Top 100 upregulated genes in ACC cells compared to normal salivary glands in the Andersson database**.** Genes ordinated for decreasing log2 fold change.

| **GENE** | **LOG2 FOLD CHANGE** | | **P VALUE** | | **P ADJ** | | **CONFIDENCE** |
| --- | --- | --- | --- | --- | --- | --- | --- |
| HORMAD1 | 0.89213783 | | 1.93E-10 | | 1.47E-08 | | **** |
| **MYB** | **0.84697945** | | **9.22E-13** | | **3.24E-10** | | ******** |
| GABRP | 0.75193462 | | 1.98E-12 | | 5.68E-10 | | **** |
| HAPLN1 | 0.74981242 | | 4.79E-07 | | 5.96E-06 | | **** |
| ABCA13 | 0.7164187 | | 2.58E-10 | | 1.87E-08 | | **** |
| BMPR1B | 0.70720871 | | 6.44E-16 | | 2.31E-12 | | **** |
| PDZK1P1 | 0.69794737 | | 4.56E-05 | | 0.00025582 | | **** |
| PDZK1 | 0.69794737 | | 4.56E-05 | | 0.00025582 | | **** |
| ART3 | 0.6681816 | | 5.02E-07 | | 6.18E-06 | | **** |
| NETO2 | 0.66548999 | | 1.96E-08 | | 4.81E-07 | | **** |
| ZNF730 | 0.66093781 | | 2.17E-12 | | 6.01E-10 | | **** |
| RN7SKP240 | 0.65611984 | | 1.40E-11 | | 2.31E-09 | | **** |
| PRAME | 0.65202058 | | 1.86E-14 | | 1.83E-11 | | **** |
| FABP7 | 0.64089862 | | 1.36E-14 | | 1.58E-11 | | **** |
| ELAVL2 | 0.63904645 | | 5.90E-11 | | 6.28E-09 | | **** |
| TBX22 | 0.63743853 | | 1.72E-11 | | 2.71E-09 | | **** |
| ANLN | 0.63258511 | | 1.15E-14 | | 1.46E-11 | | **** |
| RNA5SP101 | 0.62033523 | | 4.54E-09 | | 1.53E-07 | | **** |
| RNU6-302P | 0.60245845 | | 1.05E-09 | | 5.40E-08 | | **** |
| RNU6-446P | 0.59408654 | | 3.02E-10 | | 2.06E-08 | | **** |
| OR2L8 | 0.58621485 | | 5.68E-07 | | 6.86E-06 | | **** |
| ZNF726 | 0.57899202 | | 6.11E-12 | | 1.39E-09 | | **** |
| CDK1 | 0.57675683 | | 9.97E-10 | | 5.21E-08 | | **** |
| VTCN1 | 0.57362768 | | 6.75E-15 | | 9.06E-12 | | **** |
| BRIP1 | 0.57051645 | | 5.93E-12 | | 1.36E-09 | | **** |
| FNDC1 | 0.56992441 | | 2.79E-09 | | 1.07E-07 | | **** |
| OR2L2 | 0.56835149 | | 1.87E-07 | | 2.80E-06 | | **** |
| OR2AK2 | 0.56558098 | | 2.98E-07 | | 4.06E-06 | | **** |
| DTL | 0.55866954 | | 4.71E-10 | | 2.88E-08 | | **** |
| HELLS | 0.54835335 | | 9.36E-14 | | 6.45E-11 | | **** |
| SNORA72 | 0.54674306 | | 3.66E-06 | | 3.10E-05 | | **** |
| LINC01667 | 0.5370638 | | 2.22E-05 | | 0.00014083 | | **** |
| VCAN | 0.52995838 | | 2.67E-12 | | 6.99E-10 | | **** |
| KIF11 | 0.5194619 | | 1.23E-10 | | 1.03E-08 | | **** |
| OR2L5 | 0.51700674 | | 2.59E-06 | | 2.31E-05 | | **** |
| SHC4 | 0.51061181 | | 3.67E-09 | | 1.32E-07 | | **** |
| DLX5 | 0.50523046 | | 3.03E-06 | | 2.64E-05 | | **** |
| SYCP2 | 0.50348588 | | 1.17E-10 | | 9.86E-09 | | **** |
| CCNB2 | 0.49988228 | | 8.20E-11 | | 7.75E-09 | | **** |
| XRCC2 | 0.49680259 | | 4.27E-11 | | 5.07E-09 | | **** |
| TOP2A | 0.49585189 | | 5.34E-11 | | 5.92E-09 | | **** |
| CENPF | 0.49573719 | | 3.26E-10 | | 2.14E-08 | | **** |
| TTK | 0.49382075 | | 2.08E-08 | | 4.99E-07 | | **** |
| RNA5SP157 | 0.48560484 | | 5.93E-08 | | 1.14E-06 | | **** |
| CENPK | 0.48510544 | | 2.07E-09 | | 8.67E-08 | | **** |
| RNU6-637P | 0.48018087 | | 0.00019502 | | 0.00088035 | | *** |
| NUSAP1 | 0.47659492 | | 2.86E-10 | | 1.99E-08 | | **** |
| CENPU | 0.47229991 | | 1.09E-11 | | 1.98E-09 | | **** |
| KRT15 | 0.46765698 | | 3.89E-06 | | 3.26E-05 | | **** |
| ZNF300 | 0.46615317 | | 1.32E-12 | | 4.16E-10 | | **** |
| RAPGEF4 | 0.4658005 | | 1.94E-07 | | 2.88E-06 | | **** |
| RNU6-674P | 0.46506125 | | 1.07E-09 | | 5.46E-08 | | **** |
| DUXAP10 | 0.46337824 | | 4.99E-12 | | 1.17E-09 | | **** |
| PTH2R | 0.46190645 | | 0.00250718 | | 0.00761351 | | ** |
| PRLR | 0.45807086 | | 2.11E-08 | | 5.04E-07 | | **** |
| ADGRV1 | 0.45402419 | | 0.00011583 | | 0.00056598 | | **** |
| PCLAF | 0.45280212 | | 1.60E-08 | | 4.18E-07 | | **** |
| RPS24 | 0.45279077 | | 2.85E-06 | | 2.50E-05 | | **** |
| OBP2B | 0.45119868 | | 1.26E-07 | | 2.04E-06 | | **** |
| GUCY1A1 | 0.4493344 | | 3.83E-09 | | 1.35E-07 | | **** |
| GINS1 | 0.44704048 | | 2.47E-12 | | 6.56E-10 | | **** |
| RASGRP1 | 0.44686369 | | 7.16E-08 | | 1.31E-06 | | **** |
| SLC12A1 | 0.44374509 | | 2.22E-05 | | 0.00014062 | | **** |
| EZH2 | 0.44263498 | | 3.53E-15 | | 5.37E-12 | | **** |
| NLN | 0.44243006 | | 1.38E-12 | | 4.28E-10 | | **** |
| BUB1B | 0.44168495 | | 7.53E-11 | | 7.30E-09 | | **** |
| RNU6-1003P | 0.43816692 | | 1.20E-07 | | 1.96E-06 | | **** |
| KIF14 | 0.43764569 | | 9.48E-09 | | 2.77E-07 | | **** |
| LINC02487 | 0.437522 | | 0.00030225 | | 0.00127228 | | *** |
| POLE2 | 0.43697368 | | 1.18E-10 | | 9.92E-09 | | **** |
| HEY2 | 0.43622312 | | 1.39E-06 | | 1.39E-05 | | **** |
| TDRD12 | 0.4347428 | | 0.0093138 | | 0.02283094 | | ** |
| MELK | 0.43345808 | | 3.12E-10 | | 2.09E-08 | | **** |
| TPX2 | 0.43237384 | | 1.95E-09 | | 8.32E-08 | | **** |
| EDIL3 | 0.43192282 | | 1.32E-07 | | 2.12E-06 | | **** |
| DKK1 | 0.4247637 | | 0.00015132 | | 0.0007096 | | *** |
| GLYATL2 | 0.42030902 | | 0.00032704 | | 0.00135892 | | *** |
| ZNF286A | 0.41968677 | | 2.77E-13 | | 1.36E-10 | | **** |
| ZNF286B | 0.41968677 | | 2.77E-13 | | 1.36E-10 | | **** |
| ZNF286A-TBC1D26 | 0.41968677 | | 2.77E-13 | | 1.36E-10 | | **** |
| KNL1 | 0.41857955 | | 3.54E-09 | | 1.29E-07 | | **** |
| RN7SL73P | 0.4182692 | | 0.0003982 | | 0.0015985 | | *** |
| RPL24P8 | 0.4182692 | | 0.0003982 | | 0.0015985 | | *** |
| RPL24 | 0.4182692 | | 0.0003982 | | 0.0015985 | | *** |
| SKA3 | 0.41786658 | | 2.05E-08 | | 4.95E-07 | | **** |
| SERPINE2 | 0.41756123 | | 9.19E-06 | | 6.73E-05 | | **** |
| SHCBP1 | 0.41625138 | | 9.42E-08 | | 1.63E-06 | | **** |
| RNA5SP346 | 0.41565498 | | 0.00098562 | | 0.00343766 | | *** |
| AADAT | 0.41543912 | | 1.98E-08 | | 4.85E-07 | | **** |
| ASPN | 0.41516045 | | 0.00250235 | | 0.00760156 | | ** |
| TMSB15B | 0.41471215 | | 2.97E-09 | | 1.12E-07 | | **** |
| PLK4 | 0.41459754 | | 9.70E-11 | | 8.58E-09 | | **** |
| VIT | | 0.41412027 | | 6.17E-05 | | 0.00033199 | **** |
| CKS2 | | 0.41198799 | | 8.04E-09 | | 2.41E-07 | **** |
| CENPI | | 0.41082475 | | 1.68E-09 | | 7.64E-08 | **** |
| DIAPH3 | | 0.41072892 | | 2.92E-10 | | 2.01E-08 | **** |
| MKI67 | | 0.41004835 | | 3.48E-09 | | 1.27E-07 | **** |
| PARPBP | | 0.40977352 | | 1.97E-09 | | 8.35E-08 | **** |
| FAM111B | | 0.40871178 | | 2.17E-08 | | 5.19E-07 | **** |
| ST3GAL4 | | 0.40743091 | | 6.66E-11 | | 6.66E-09 | **** |
| MND1 | | 0.40569555 | | 1.32E-09 | | 6.39E-08 | **** |
| CDK6 | | 0.40366537 | | 1.14E-13 | | 7.62E-11 | **** |
| PBK | | 0.40316531 | | 2.21E-07 | | 3.21E-06 | **** |
| KIF23 | | 0.40234105 | | 1.13E-11 | | 2.00E-09 | **** |
| ZNF571-AS1 | | 0.40167358 | | 1.97E-05 | | 0.00012858 | **** |
| NUF2 | | 0.40094437 | | 3.29E-09 | | 1.22E-07 | **** |
| DLGAP5 | | 0.39899149 | | 1.69E-08 | | 4.37E-07 | **** |
| KIF20A | | 0.39875618 | | 3.96E-10 | | 2.51E-08 | **** |
| CDCA7 | | 0.39873082 | | 1.69E-10 | | 1.34E-08 | **** |
| BUB1 | | 0.39810605 | | 1.88E-10 | | 1.44E-08 | **** |
| BAMBI | | 0.39617182 | | 8.58E-06 | | 6.34E-05 | **** |
| TMEFF1 | | 0.39501737 | | 2.47E-11 | | 3.48E-09 | **** |
| MSANTD3-TMEFF1 | | 0.39501737 | | 2.47E-11 | | 3.48E-09 | **** |
| LINC00885 | | 0.39464798 | | 2.34E-11 | | 3.34E-09 | **** |
| POGLUT2 | | 0.39334692 | | 1.14E-10 | | 9.66E-09 | **** |
| SEPTIN4 | | 0.39325391 | | 2.47E-10 | | 1.80E-08 | **** |
| THBS2 | | 0.39232335 | | 3.49E-08 | | 7.50E-07 | **** |
| ESCO2 | | 0.39109836 | | 1.25E-07 | | 2.03E-06 | **** |
| MAMDC2 | | 0.38951529 | | 0.0005473 | | 0.00208999 | *** |
| EPHA7 | | 0.38894995 | | 6.67E-07 | | 7.81E-06 | **** |
| PXDN | | 0.3881627 | | 8.92E-08 | | 1.55E-06 | **** |
| APBA2 | | 0.38814688 | | 3.82E-10 | | 2.44E-08 | **** |
| NPNT | | 0.38666574 | | 2.82E-09 | | 1.08E-07 | **** |
| COL9A1 | | 0.38666508 | | 1.60E-05 | | 0.00010771 | **** |
| LINC00665 | | 0.38625975 | | 1.04E-09 | | 5.37E-08 | **** |
| LINC01535 | | 0.38625975 | | 1.04E-09 | | 5.37E-08 | **** |
| FAM227A | | 0.38469431 | | 9.63E-11 | | 8.57E-09 | **** |
| ASPM | | 0.38375044 | | 1.59E-09 | | 7.35E-08 | **** |
| UBE2T | | 0.38369793 | | 4.44E-10 | | 2.74E-08 | **** |
| ENC1 | | 0.38343682 | | 1.13E-10 | | 9.65E-09 | **** |
| TTYH1 | | 0.3833019 | | 1.35E-09 | | 6.49E-08 | **** |
| ARHGAP11A | | 0.38243442 | | 1.50E-08 | | 4.00E-07 | **** |
| KIF15 | | 0.38189479 | | 4.88E-08 | | 9.75E-07 | **** |
| PLCH1 | | 0.38068003 | | 6.98E-06 | | 5.31E-05 | **** |
| POSTN | | 0.38002935 | | 0.02406732 | | 0.05093338 | * |
| SGO1 | | 0.37981864 | | 1.17E-06 | | 1.20E-05 | **** |
| FANCI | | 0.37537925 | | 1.81E-09 | | 7.99E-08 | **** |
| COLEC12 | | 0.37525028 | | 6.47E-07 | | 7.64E-06 | **** |
| CENPE | | 0.37510122 | | 3.29E-08 | | 7.14E-07 | **** |
| ST8SIA6 | | 0.37156426 | | 0.00196356 | | 0.00619437 | ** |
| ZNF682 | | 0.37136551 | | 6.99E-11 | | 6.89E-09 | **** |
| PIEZO2 | | 0.37096309 | | 0.00681374 | | 0.01762969 | ** |
| H2BC7 | | 0.36877212 | | 4.63E-07 | | 5.79E-06 | **** |
| DUXAP8 | | 0.36808479 | | 6.43E-12 | | 1.43E-09 | **** |
| RPS25 | | 0.36773215 | | 0.00103338 | | 0.00357635 | *** |
| WDHD1 | | 0.36665549 | | 8.39E-11 | | 7.87E-09 | **** |
| SEMA6D | | 0.36665151 | | 2.00E-08 | | 4.87E-07 | **** |
| NCAPG | | 0.3665404 | | 2.69E-09 | | 1.04E-07 | **** |
| CERS3 | | 0.36539466 | | 0.00225607 | | 0.00696567 | ** |
| RAD51AP1 | 0.36381966 | | 1.35E-08 | | 3.68E-07 | | **** |

**Supplementary Table 4.** Top 150 upregulated genes in ACC cells compared to normal salivary glands in the Chowbina database**.** Genes were ordinated for decreasing log2 fold change.

| **GENE** | **LOG2 FOLD CHANGE** | | **P VALUE** | | **P ADJ** | | **CONFIDENCE** |
| --- | --- | --- | --- | --- | --- | --- | --- |
| EN1 | 6.04059354 | | 3.01E-12 | | 1.64E-09 | | **** |
| FABP7 | 5.92613776 | | 1.67E-08 | | 2.45E-06 | | **** |
| IGF2 | 5.61381622 | | 5.07E-05 | | 0.00104071 | | **** |
| **MYB** | **5.41311003** | | **4.60E-05** | | **0.00096597** | | ******** |
| FNDC1 | 5.09886147 | | 1.55E-09 | | 3.42E-07 | | **** |
| VCAN | 4.99819954 | | 5.66E-06 | | 0.00021992 | | **** |
| IGFBP2 | 4.99606766 | | 4.53E-06 | | 0.00018731 | | **** |
| RRM2 | 4.94537289 | | 4.96E-08 | | 5.99E-06 | | **** |
| COL27A1 | 4.82953275 | | 1.07E-07 | | 1.09E-05 | | **** |
| NUSAP1 | 4.7867362 | | 3.55E-08 | | 4.66E-06 | | **** |
| HAPLN1 | 4.68972911 | | 0.00633487 | | 0.03772405 | | ** |
| GABRP | 4.60316414 | | 2.25E-07 | | 1.98E-05 | | **** |
| VTCN1 | 4.59027596 | | 5.43E-08 | | 6.39E-06 | | **** |
| SOX11 | 4.49325499 | | 0.00909669 | | 0.04911993 | | ** |
| PCLAF | 4.43466531 | | 7.11E-08 | | 8.11E-06 | | **** |
| HORMAD1 | 4.30752547 | | 1.03E-06 | | 6.20E-05 | | **** |
| PDZK1 | 4.29216281 | | 0.02788633 | | 0.10999357 | | * |
| PDZK1P1 | 4.29216281 | | 0.02788633 | | 0.10999357 | | * |
| LAMB1 | 4.2180471 | | 1.77E-08 | | 2.59E-06 | | **** |
| CKS2 | 4.13922976 | | 5.54E-09 | | 9.42E-07 | | **** |
| TTYH1 | 4.13722216 | | 0.00010515 | | 0.00180266 | | **** |
| DTL | 4.11459974 | | 7.75E-07 | | 5.08E-05 | | **** |
| TOP2A | 4.10106158 | | 6.28E-07 | | 4.38E-05 | | **** |
| ASPM | 4.08522542 | | 7.57E-07 | | 4.99E-05 | | **** |
| PNMA8A | 4.07222369 | | 5.47E-05 | | 0.00110669 | | **** |
| CDCA7 | 4.03277266 | | 2.18E-08 | | 3.07E-06 | | **** |
| PRAME | 4.01045853 | | 7.86E-08 | | 8.74E-06 | | **** |
| PBK | 3.98652301 | | 4.04E-06 | | 0.00016974 | | **** |
| MIR675 | 3.97226768 | | 0.02253108 | | 0.09480187 | | * |
| NRCAM | 3.96362984 | | 1.43E-08 | | 2.15E-06 | | **** |
| EZH2 | 3.9308591 | | 6.53E-07 | | 4.53E-05 | | **** |
| ART3 | 3.93005526 | | 1.35E-05 | | 0.00041026 | | **** |
| SOX4 | 3.88032844 | | 3.42E-08 | | 4.52E-06 | | **** |
| BASP1 | 3.81780376 | | 2.33E-08 | | 3.26E-06 | | **** |
| CENPU | 3.80269213 | | 1.73E-06 | | 8.72E-05 | | **** |
| KIF11 | 3.80062968 | | 1.15E-07 | | 1.16E-05 | | **** |
| LGR6 | 3.77654375 | | 0.0004709 | | 0.00553466 | | *** |
| SERPINE2 | 3.77151196 | | 1.49E-05 | | 0.00044003 | | **** |
| DLGAP5 | 3.77014347 | | 4.09E-06 | | 0.00017135 | | **** |
| TTK | 3.74170154 | | 7.35E-06 | | 0.00026533 | | **** |
| COL11A1 | 3.73140278 | | 0.00161422 | | 0.0137943 | | *** |
| COL9A2 | 3.7159813 | | 1.23E-05 | | 0.00038586 | | **** |
| MIR483 | 3.70854505 | | 0.00321259 | | 0.02294151 | | ** |
| INS-IGF2 | 3.70854505 | | 0.00321259 | | 0.02294151 | | ** |
| MFAP2 | 3.70441833 | | 0.00046845 | | 0.00550882 | | *** |
| GINS1 | 3.70114619 | | 2.15E-06 | | 0.00010252 | | **** |
| CDC7 | 3.69875565 | | 0.00017337 | | 0.00264558 | | *** |
| PRC1 | 3.6680321 | | 2.45E-07 | | 2.12E-05 | | **** |
| KIF20A | 3.66601867 | | 1.15E-06 | | 6.68E-05 | | **** |
| NETO2 | 3.66061385 | | 0.00390977 | | 0.02646169 | | ** |
| MELK | 3.63689514 | | 1.26E-07 | | 1.23E-05 | | **** |
| NPNT | 3.62746331 | | 0.00245824 | | 0.01893654 | | ** |
| BUB1B | 3.60805284 | | 2.88E-06 | | 0.00012872 | | **** |
| SLC35F3 | 3.6008696 | | 0.00011951 | | 0.00197506 | | **** |
| ANLN | 3.5831178 | | 3.10E-07 | | 2.58E-05 | | **** |
| E2F7 | 3.56483462 | | 1.07E-06 | | 6.39E-05 | | **** |
| ZWINT | 3.56046338 | | 7.41E-07 | | 4.94E-05 | | **** |
| TMSB15A | 3.54688019 | | 6.53E-05 | | 0.00128037 | | **** |
| LINC01139 | 3.52653143 | | 0.00259024 | | 0.01969497 | | ** |
| DLX2 | 3.50356647 | | 0.00828964 | | 0.04585868 | | ** |
| CENPK | 3.49927126 | | 0.00020931 | | 0.00305425 | | *** |
| HMMR | 3.47450946 | | 3.39E-07 | | 2.77E-05 | | **** |
| WDFY2 | 3.46571814 | | 1.97E-06 | | 9.56E-05 | | **** |
| FAM83D | 3.45542458 | | 5.38E-06 | | 0.00021319 | | **** |
| ZNF300 | 3.44476881 | | 7.90E-08 | | 8.74E-06 | | **** |
| PCDHB10 | 3.42607489 | | 2.46E-05 | | 0.00062702 | | **** |
| SCG5 | 3.41347004 | | 0.01264584 | | 0.06246084 | | ** |
| ARHGAP11A-SCG5 | 3.41347004 | | 0.01264584 | | 0.06246084 | | ** |
| EFHD1 | 3.39496915 | | 1.87E-10 | | 6.34E-08 | | **** |
| OBP2B | 3.37606868 | | 0.00011448 | | 0.001907 | | **** |
| CDK1 | 3.37282853 | | 5.89E-06 | | 0.00022538 | | **** |
| MTHFD1L | 3.29704266 | | 1.27E-06 | | 7.06E-05 | | **** |
| MIR100HG | 3.29660651 | | 0.00025216 | | 0.00349687 | | *** |
| CDC20 | 3.25868422 | | 1.46E-05 | | 0.00043311 | | **** |
| TP53 | 3.24665758 | | 7.83E-10 | | 1.97E-07 | | **** |
| MOK | 3.22153185 | | 3.26E-05 | | 0.00075569 | | **** |
| ZNF367 | 3.20270786 | | 1.13E-06 | | 6.62E-05 | | **** |
| TMEM100 | 3.18710086 | | 0.01985127 | | 0.08646883 | | * |
| NDC80 | 3.18599734 | | 3.98E-05 | | 0.00086219 | | **** |
| RAD51AP1 | 3.18087398 | | 2.37E-06 | | 0.00010933 | | **** |
| BAMBI | 3.15996619 | | 2.83E-05 | | 0.00067893 | | **** |
| SPARC | 3.15974268 | | 1.94E-06 | | 9.48E-05 | | **** |
| UBE2SP1 | 3.13970659 | | 8.33E-08 | | 9.04E-06 | | **** |
| UBE2S | 3.13970659 | | 8.33E-08 | | 9.04E-06 | | **** |
| UBE2SP2 | 3.13970659 | | 8.33E-08 | | 9.04E-06 | | **** |
| PDE9A | 3.1360691 | | 5.64E-09 | | 9.48E-07 | | **** |
| CCNB1 | 3.13129911 | | 1.80E-05 | | 0.00050555 | | **** |
| ZNF727 | 3.11555949 | | 0.00018039 | | 0.0027339 | | *** |
| MIA | 3.11325772 | | 7.30E-05 | | 0.0013835 | | **** |
| MIA-RAB4B | 3.11325772 | | 7.30E-05 | | 0.0013835 | | **** |
| PRELP | 3.10945043 | | 0.00425019 | | 0.0281494 | | ** |
| MLC1 | 3.10885397 | | 0.0056868 | | 0.03477257 | | ** |
| HELLS | | 3.09247353 | | 1.03E-05 | | 0.00034 | **** |
| COLEC12 | | 3.08856344 | | 0.00133036 | | 0.01203568 | *** |
| TPX2 | | 3.08716842 | | 1.19E-05 | | 0.00037688 | **** |
| IGDCC4 | | 3.084614 | | 0.00067735 | | 0.00719583 | *** |
| RACGAP1 | | 3.05240595 | | 8.79E-06 | | 0.00030475 | **** |
| RFLNA | | 3.05082202 | | 0.00051716 | | 0.00592726 | *** |
| NEK2 | | 3.04867288 | | 0.0001136 | | 0.00189501 | **** |
| NUDT11 | | 3.04350644 | | 6.95E-07 | | 4.75E-05 | **** |
| TMEFF1 | | 3.03636107 | | 0.0025554 | | 0.01949024 | ** |
| MSANTD3-TMEFF1 | | 3.03636107 | | 0.0025554 | | 0.01949024 | ** |
| MARCKSL1 | | 3.00481511 | | 0.00052726 | | 0.00602292 | *** |
| SOX8 | | 3.00059656 | | 0.00221801 | | 0.01752689 | ** |
| KRT5 | | 2.99681887 | | 3.42E-09 | | 6.24E-07 | **** |
| SHC4 | | 2.98719611 | | 0.00604698 | | 0.03641304 | ** |
| SERPINH1 | | 2.97049972 | | 3.20E-06 | | 0.0001408 | **** |
| CENPA | | 2.96717264 | | 7.86E-06 | | 0.0002785 | **** |
| CEP55 | | 2.96234642 | | 1.71E-05 | | 0.00049046 | **** |
| PLAT | | 2.95677382 | | 0.07325691 | | 0.21491578 | ns |
| NRTN | | 2.95656007 | | 0.00011811 | | 0.00195873 | **** |
| SCHIP1 | | 2.95566249 | | 5.76E-06 | | 0.00022243 | **** |
| IQCJ-SCHIP1 | | 2.95566249 | | 5.76E-06 | | 0.00022243 | **** |
| VIT | | 2.95561732 | | 0.00788994 | | 0.04427196 | ** |
| DKK1 | | 2.95546573 | | 0.01669769 | | 0.07638998 | ** |
| ZNF286B | | 2.94085566 | | 1.94E-06 | | 9.48E-05 | **** |
| ZNF286A | | 2.94085566 | | 1.94E-06 | | 9.48E-05 | **** |
| CDKN3 | | 2.93944554 | | 2.57E-06 | | 0.00011653 | **** |
| NCKAP5 | | 2.93303902 | | 4.59E-05 | | 0.00096597 | **** |
| KRT12 | | 2.93173806 | | 0.04449997 | | 0.15309277 | * |
| POLE2 | | 2.92169548 | | 5.81E-06 | | 0.00022408 | **** |
| FLVCR1 | | 2.91623057 | | 1.55E-06 | | 8.12E-05 | **** |
| EFS | | 2.91231541 | | 1.16E-07 | | 1.16E-05 | **** |
| SCRG1 | | 2.90966891 | | 0.00039745 | | 0.00486584 | *** |
| MCM10 | | 2.90836332 | | 1.04E-05 | | 0.0003449 | **** |
| UHRF1 | | 2.90710256 | | 0.00035521 | | 0.00449895 | *** |
| SUSD5 | | 2.90044996 | | 0.02258627 | | 0.09499831 | * |
| NPTX2 | | 2.88978845 | | 0.02776691 | | 0.10970825 | * |
| KIF2C | | 2.87270536 | | 3.78E-07 | | 3.01E-05 | **** |
| PCSK1N | | 2.8713662 | | 4.90E-06 | | 0.00019921 | **** |
| TMEM158 | | 2.86522736 | | 0.00021382 | | 0.00310939 | *** |
| COL4A2 | | 2.84469114 | | 0.00057328 | | 0.00639824 | *** |
| EPHX4 | | 2.8370785 | | 3.53E-05 | | 0.00079568 | **** |
| NRARP | | 2.83026664 | | 1.60E-05 | | 0.00046481 | **** |
| NUF2 | | 2.82815546 | | 0.00019389 | | 0.00288389 | *** |
| MIR106B | | 2.82369977 | | 1.80E-09 | | 3.76E-07 | **** |
| MCM7 | | 2.82369977 | | 1.80E-09 | | 3.76E-07 | **** |
| MIR25 | | 2.82369977 | | 1.80E-09 | | 3.76E-07 | **** |
| MIR93 | | 2.82369977 | | 1.80E-09 | | 3.76E-07 | **** |
| DLX5 | | 2.82233995 | | 0.01971256 | | 0.086112 | ** |
| FANCI | | 2.8196455 | | 6.06E-06 | | 0.00022987 | **** |
| CDC25B | | 2.81522436 | | 1.80E-05 | | 0.00050555 | **** |
| KIF15 | | 2.80024906 | | 0.00036498 | | 0.00458521 | *** |
| TMEM97 | | 2.79835354 | | 9.82E-06 | | 0.00032778 | **** |
| CDH2 | | 2.78468037 | | 0.01966061 | | 0.08597672 | ** |
| DAPK1 | | 2.78198067 | | 7.19E-05 | | 0.00136894 | **** |
| KIF14 | | 2.77629605 | | 0.0001896 | | 0.00284839 | *** |
| PCDHB14 | | 2.77165495 | | 0.01677374 | | 0.07667324 | ** |
| H2AX | | 2.76892465 | | 4.56E-06 | | 0.00018818 | **** |
| CARM1 | 2.76076058 | | 1.65E-06 | | 8.44E-05 | | **** |

**Supplementary Table 5.** Top 150 upregulated genes in ACC cells compared to normal salivary glands in the Chowbina database**.** Genes were ordinated for decreasing log2 fold change.

| **GENE** | **LOG2 FOLD CHANGE** | **P VALUE** | **P ADJ** | **CONFIDENCE** |
| --- | --- | --- | --- | --- |
| ABCA13 | 13.9564246 | 3.34E-29 | 7.72E-28 | **** |
| PRAME | 13.8908994 | 1.64E-30 | 4.04E-29 | **** |
| EN1 | 12.9844084 | 3.17E-27 | 6.56E-26 | **** |
| NKX6-1 | 12.5413661 | 2.03E-23 | 3.29E-22 | **** |
| LINC02582 | 12.1776121 | 5.31E-23 | 8.36E-22 | **** |
| OBP2B | 11.9343229 | 7.45E-24 | 1.24E-22 | **** |
| LINC01505 | 11.8911723 | 7.34E-22 | 1.06E-20 | **** |
| SLC6A10P | 11.5735034 | 1.80E-21 | 2.53E-20 | **** |
| ZFP42 | 11.5684743 | 2.11E-22 | 3.16E-21 | **** |
| FNDC1 | 11.4453421 | 4.42E-19 | 5.21E-18 | **** |
| DIPK1C | 11.2514162 | 8.59E-17 | 8.66E-16 | **** |
| LIX1 | 11.2487388 | 1.51E-12 | 1.08E-11 | **** |
| C6orf15 | 11.1727149 | 1.68E-10 | 9.82E-10 | **** |
| HORMAD1 | 11.0854739 | 5.49E-20 | 6.89E-19 | **** |
| KRT6B | 10.9592223 | 8.10E-93 | 2.07E-90 | **** |
| IGFL1 | 10.9424806 | 1.73E-16 | 1.70E-15 | **** |
| CRABP1 | 10.9079558 | 2.94E-12 | 2.04E-11 | **** |
| SOX11 | 10.8778313 | 1.10E-19 | 1.34E-18 | **** |
| HOXC13 | 10.8544446 | 2.69E-19 | 3.21E-18 | **** |
| SIX3 | 10.8428501 | 1.45E-12 | 1.03E-11 | **** |
| ROS1 | 10.6514316 | 1.70E-09 | 9.00E-09 | **** |
| PURPL | 10.4353249 | 5.04E-17 | 5.19E-16 | **** |
| COL2A1 | 10.233195 | 2.09E-22 | 3.14E-21 | **** |
| KIF14 | 10.2204062 | 4.39E-16 | 4.19E-15 | **** |
| POU6F2 | 10.1248193 | 1.84E-16 | 1.80E-15 | **** |
| ASPM | 10.1091264 | 8.98E-16 | 8.39E-15 | **** |
| S100P | 10.1068906 | 5.13E-33 | 1.44E-31 | **** |
| CLSPN | 10.0334145 | 1.44E-16 | 1.43E-15 | **** |
| ANKRD1 | 10.0224725 | 1.88E-35 | 6.16E-34 | **** |
| FGFBP1 | 9.99883461 | 5.26E-14 | 4.24E-13 | **** |
| EPHX4 | 9.98005645 | 4.48E-16 | 4.27E-15 | **** |
| SCGB1A1 | 9.96107553 | 1.63E-07 | 6.86E-07 | **** |
| KIF4A | 9.93345548 | 1.62E-15 | 1.48E-14 | **** |
| DLX2 | 9.89103733 | 4.17E-13 | 3.14E-12 | **** |
| GABRA5 | 9.86737883 | 2.50E-15 | 2.25E-14 | **** |
| ANLN | 9.8596671 | 8.93E-48 | 5.17E-46 | **** |
| MELK | 9.8000806 | 3.21E-15 | 2.86E-14 | **** |
| FOSL1 | 9.74861276 | 1.54E-19 | 1.87E-18 | **** |
| PWP2 | 9.63499795 | 9.34E-16 | 8.71E-15 | **** |
| CDCA2 | 9.58396167 | 3.21E-15 | 2.86E-14 | **** |
| KRT12 | 9.56315133 | 7.09E-15 | 6.15E-14 | **** |
| LINC01198 | 9.52849271 | 4.74E-09 | 2.40E-08 | **** |
| CENPF | 9.34448034 | 1.45E-40 | 6.00E-39 | **** |
| ELAVL2 | 9.26374384 | 1.11E-12 | 8.01E-12 | **** |
| LCN1P1 | 9.20506948 | 1.52E-08 | 7.24E-08 | **** |
| QRFPR | 9.19676686 | 3.27E-12 | 2.26E-11 | **** |
| RPL23AP21 | 9.13264927 | 9.17E-14 | 7.24E-13 | **** |
| TOP2A | 9.12789276 | 4.16E-54 | 3.03E-52 | **** |
| KRT6A | 9.09671881 | 1.29E-18 | 1.48E-17 | **** |
| MIR3187 | 9.0563497 | 1.57E-12 | 1.12E-11 | **** |
| HOXC13-AS | 9.04117574 | 2.24E-13 | 1.72E-12 | **** |
| SOX14 | 9.03572689 | 3.79E-08 | 1.72E-07 | **** |
| THEG | 8.98801231 | 7.22E-13 | 5.30E-12 | **** |
| LINC02899 | 8.909893 | 1.18E-13 | 9.26E-13 | **** |
| MKI67 | 8.88158556 | 8.75E-60 | 7.78E-58 | **** |
| CENPE | 8.84905845 | 5.18E-16 | 4.92E-15 | **** |
| MYBL2 | 8.83218706 | 4.02E-22 | 5.90E-21 | **** |
| TAGLN3 | 8.79666382 | 1.04E-12 | 7.50E-12 | **** |
| EPIC1 | 8.79296075 | 7.29E-13 | 5.35E-12 | **** |
| INA | 8.78490304 | 4.12E-13 | 3.10E-12 | **** |
| CLPSL1 | 8.76012289 | 3.08E-06 | 1.11E-05 | **** |
| CENPVL3 | 8.75986696 | 2.05E-11 | 1.31E-10 | **** |
| PLPPR3 | 8.69583889 | 1.54E-38 | 5.81E-37 | **** |
| ZIC1 | 8.64550129 | 2.42E-12 | 1.69E-11 | **** |
| NEIL3 | 8.58829341 | 8.79E-11 | 5.29E-10 | **** |
| DSG3 | 8.57295492 | 4.38E-76 | 6.28E-74 | **** |
| LINC01291 | 8.5694891 | 7.96E-13 | 5.82E-12 | **** |
| NKX2-5 | 8.52355985 | 4.62E-29 | 1.06E-27 | **** |
| FOXI3 | 8.4243172 | 1.05E-28 | 2.36E-27 | **** |
| LINC00602 | 8.41974289 | 4.81E-12 | 3.26E-11 | **** |
| HS6ST2 | 8.35986544 | 2.70E-49 | 1.65E-47 | **** |
| NTSR1 | 8.35470114 | 1.52E-08 | 7.22E-08 | **** |
| KC6 | 8.34610105 | 4.99E-11 | 3.08E-10 | **** |
| ADAMTS16-DT | 8.34058504 | 6.00E-35 | 1.89E-33 | **** |
| BBOX1 | 8.28796944 | 9.04E-14 | 7.15E-13 | **** |
| GABRP | 8.25693949 | 0 | 0 | **** |
| OBP2A | 8.22210565 | 7.00E-12 | 4.68E-11 | **** |
| KRT16 | 8.21710678 | 4.17E-72 | 5.52E-70 | **** |
| CLPS | 8.20056733 | 9.93E-06 | 3.34E-05 | **** |
| ACTBL2 | 8.13420358 | 9.02E-09 | 4.42E-08 | **** |
| KRT6C | 8.13293952 | 1.19E-17 | 1.28E-16 | **** |
| VAX1 | 8.12392392 | 4.14E-09 | 2.11E-08 | **** |
| BUB1B | 8.09862606 | 2.96E-22 | 4.38E-21 | **** |
| MLC1 | 8.07540535 | 1.30E-36 | 4.50E-35 | **** |
| PRSS3 | 8.05636491 | 0.00021358 | 0.00058595 | *** |
| KIF18B | 8.03934252 | 4.16E-21 | 5.69E-20 | **** |
| LNCAROD | 8.01825938 | 1.99E-07 | 8.31E-07 | **** |
| HOXC10 | 8.00756122 | 1.57E-10 | 9.23E-10 | **** |
| CXCL5 | 8.00310444 | 8.65E-15 | 7.44E-14 | **** |
| ALOX12B | 7.99134089 | 5.96E-11 | 3.66E-10 | **** |
| CCNA1 | 7.9894491 | 1.17E-23 | 1.92E-22 | **** |
| CYP1B1 | 7.96494345 | 1.48E-15 | 1.36E-14 | **** |
| GABBR2 | 7.95315046 | 1.84E-10 | 1.07E-09 | **** |
| CD109-AS1 | 7.92675114 | 1.99E-10 | 1.16E-09 | **** |
| CTNND2 | 7.92476109 | 1.93E-26 | 3.79E-25 | **** |
| LINC02275 | 7.90162006 | 2.37E-09 | 1.24E-08 | **** |
| KIAA1549L | 7.90084587 | 2.07E-37 | 7.45E-36 | **** |
| TLX1 | 7.84555546 | 5.42E-10 | 3.01E-09 | **** |
| LINC00461 | 7.82739155 | 1.71E-21 | 2.40E-20 | **** |
| SKA1 | 7.79510515 | 5.12E-10 | 2.85E-09 | **** |
| ZIC4 | 7.7782175 | 1.96E-10 | 1.14E-09 | **** |
| PBK | 7.77401588 | 1.82E-22 | 2.75E-21 | **** |
| BUB1 | 7.73484696 | 4.89E-23 | 7.72E-22 | **** |
| TPRXL | 7.72029795 | 6.33E-09 | 3.16E-08 | **** |
| DLL3 | 7.70513445 | 2.99E-10 | 1.71E-09 | **** |
| KRT81 | 7.62799172 | 1.51E-76 | 2.21E-74 | **** |
| AURKB | 7.62389628 | 5.86E-17 | 5.99E-16 | **** |
| LOC100131536 | 7.58181044 | 4.46E-08 | 2.01E-07 | **** |
| GBX2 | 7.5213529 | 3.30E-06 | 1.18E-05 | **** |
| AKR1B10 | 7.50591029 | 1.07E-05 | 3.58E-05 | **** |
| CKS2 | 7.47007175 | 4.12E-31 | 1.05E-29 | **** |
| UPK1B | 7.42907622 | 0.00041245 | 0.00107944 | *** |
| ZNF556 | 7.41898912 | 1.01E-23 | 1.67E-22 | **** |
| CENPA | 7.41365641 | 5.75E-11 | 3.54E-10 | **** |
| DEPDC1 | 7.41342057 | 1.60E-17 | 1.70E-16 | **** |
| PCDHA2 | 7.3933462 | 1.24E-09 | 6.64E-09 | **** |
| DLGAP5 | 7.36759602 | 1.48E-20 | 1.96E-19 | **** |
| UBE2C | 7.33215214 | 1.41E-31 | 3.69E-30 | **** |
| CPA4 | 7.3303015 | 2.18E-22 | 3.27E-21 | **** |
| PCDH8 | 7.31604507 | 9.04E-08 | 3.93E-07 | **** |
| GLDC | 7.30530423 | 1.86E-31 | 4.81E-30 | **** |
| PAEP | 7.29731216 | 3.16E-07 | 1.28E-06 | **** |
| ZNF469 | 7.29535744 | 3.72E-162 | 3.73E-159 | **** |
| HOXC11 | 7.29222566 | 3.00E-08 | 1.38E-07 | **** |
| EGR4 | 7.28906956 | 1.17E-06 | 4.46E-06 | **** |
| TPX2 | 7.28643648 | 3.65E-41 | 1.57E-39 | **** |
| ADORA2B | 7.28033575 | 1.14E-61 | 1.08E-59 | **** |
| ADAMTS16 | 7.26928801 | 5.99E-96 | 1.60E-93 | **** |
| MCM10 | 7.26823543 | 8.90E-18 | 9.68E-17 | **** |
| EPHA8 | 7.26673485 | 0.0032683 | 0.00723939 | ** |
| SKA3 | 7.24306122 | 6.74E-15 | 5.87E-14 | **** |
| UHRF1 | 7.23096123 | 2.49E-31 | 6.38E-30 | **** |
| A2ML1 | 7.20192119 | 5.59E-10 | 3.10E-09 | **** |
| DTL | 7.1960906 | 6.89E-16 | 6.49E-15 | **** |
| NDST3 | 7.18405143 | 2.49E-08 | 1.16E-07 | **** |
| SIX3-AS1 | 7.17410092 | 8.99E-06 | 3.04E-05 | **** |
| GPR158 | 7.16868821 | 2.56E-06 | 9.31E-06 | **** |
| PIEZO2 | 7.15525606 | 1.65E-19 | 2.00E-18 | **** |
| HOTAIR | 7.15096848 | 7.29E-09 | 3.62E-08 | **** |
| LOC107985075 | 7.1475596 | 8.91E-09 | 4.38E-08 | **** |
| KLK14 | 7.14719283 | 1.60E-07 | 6.77E-07 | **** |
| OR7E101P | 7.14716903 | 8.74E-06 | 2.96E-05 | **** |
| MCHR1 | 7.14541464 | 8.02E-08 | 3.50E-07 | **** |
| CTSV | 7.13646094 | 4.37E-27 | 8.95E-26 | **** |
| RIMS2 | 7.1260171 | 5.76E-08 | 2.56E-07 | **** |
| FAM83D | 7.10482093 | 1.29E-35 | 4.27E-34 | **** |
| LOC112268117 | 7.10060278 | 1.98E-06 | 7.31E-06 | **** |
| **MYB** | **7.094471** | **4.24E-33** | **1.19E-31** | ******** |
| CCDC144A | 7.08706103 | 2.03E-17 | 2.14E-16 | **** |
| KIAA1210 | 7.08535148 | 1.13E-06 | 4.32E-06 | **** |

**Supplementary Table 6.** Top 100 upregulated genes in ACC cells compared to normal salivary glands in the Gao database**.** Genes were filtered for FDR ≤ 0.05 and ordinated for decreasing log2 fold change.

| **GENE** | **LOG2 FOLD CHANGE** | **P VALUE** | **P ADJ** | **CONFIDENCE** |
| --- | --- | --- | --- | --- |
| ART3 | 0.67935827 | 2.43E-13 | 8.86E-10 | **** |
| **MYB** | **0.63042863** | **1.19E-09** | **2.83E-07** | ******** |
| SHC4 | 0.60767572 | 4.55E-11 | 2.76E-08 | **** |
| RN7SKP240 | 0.59132741 | 2.50E-15 | 2.64E-11 | **** |
| RNPS1 | 0.55532933 | 4.70E-05 | 0.00054425 | **** |
| FABP7 | 0.55436947 | 1.30E-11 | 1.22E-08 | **** |
| PDZK1 | 0.54900041 | 1.29E-06 | 3.88E-05 | **** |
| PDZK1P1 | 0.54900041 | 1.29E-06 | 3.88E-05 | **** |
| TBX22 | 0.5462117 | 2.14E-09 | 4.63E-07 | **** |
| RNU6-853P | 0.54614575 | 8.84E-08 | 6.38E-06 | **** |
| HORMAD1 | 0.53812108 | 3.40E-05 | 0.00042964 | **** |
| RNU6-637P | 0.53247878 | 1.56E-06 | 4.36E-05 | **** |
| KAT7 | 0.52131329 | 7.87E-05 | 0.0007973 | **** |
| VCAN | 0.51390686 | 1.55E-12 | 2.83E-09 | **** |
| RNU6-540P | 0.5090587 | 3.66E-07 | 1.66E-05 | **** |
| ABCA13 | 0.50776709 | 2.79E-06 | 6.74E-05 | **** |
| PRAME | 0.49965249 | 4.25E-10 | 1.35E-07 | **** |
| HAPLN1 | 0.49907447 | 4.26E-06 | 9.23E-05 | **** |
| RNU6-446P | 0.49622005 | 1.17E-07 | 7.62E-06 | **** |
| ELAVL2 | 0.48450786 | 9.35E-08 | 6.44E-06 | **** |
| FNDC1 | 0.48069652 | 4.97E-11 | 2.82E-08 | **** |
| RNA5SP101 | 0.47667856 | 2.66E-09 | 5.44E-07 | **** |
| PCDHB4 | 0.4647641 | 6.83E-07 | 2.58E-05 | **** |
| RNU6-674P | 0.46395115 | 1.33E-07 | 8.46E-06 | **** |
| IL17RB | 0.46309928 | 2.57E-10 | 9.47E-08 | **** |
| BMPR1B | 0.46307375 | 7.33E-10 | 2.08E-07 | **** |
| DNAH14 | 0.46090218 | 1.57E-07 | 9.42E-06 | **** |
| VTCN1 | 0.45335164 | 3.19E-07 | 1.51E-05 | **** |
| ITGA9 | 0.44355078 | 1.89E-14 | 1.20E-10 | **** |
| RNU6-433P | 0.44026722 | 4.56E-07 | 1.94E-05 | **** |
| ZNF730 | 0.43935682 | 3.97E-11 | 2.59E-08 | **** |
| RNU6-831P | 0.43746892 | 6.55E-08 | 5.20E-06 | **** |
| NETO2 | 0.43454259 | 5.33E-07 | 2.17E-05 | **** |
| CCDC144B | 0.43192957 | 4.40E-09 | 7.00E-07 | **** |
| CCDC144A | 0.43192957 | 4.40E-09 | 7.00E-07 | **** |
| CCDC144CP | 0.43192957 | 4.40E-09 | 7.00E-07 | **** |
| GABRP | 0.42908634 | 1.04E-07 | 7.03E-06 | **** |
| DUXAP8 | 0.42856773 | 3.11E-15 | 2.64E-11 | **** |
| SEMA6D | 0.42765534 | 2.95E-11 | 2.15E-08 | **** |
| SYCP2 | 0.42746006 | 1.87E-06 | 5.00E-05 | **** |
| PCDHB3 | 0.42333659 | 1.71E-05 | 0.00025659 | **** |
| AADAT | 0.42220992 | 7.07E-10 | 2.02E-07 | **** |
| DUXAP10 | 0.41587303 | 3.09E-15 | 2.64E-11 | **** |
| PLCL1 | 0.40891366 | 4.16E-09 | 6.89E-07 | **** |
| RNU6-302P | 0.40548902 | 7.85E-08 | 5.80E-06 | **** |
| RNU6-944P | 0.40371664 | 7.82E-06 | 0.00014146 | **** |
| LRRN1 | 0.40298417 | 2.91E-05 | 0.00038193 | **** |
| NTRK3 | 0.40120395 | 2.67E-10 | 9.47E-08 | **** |
| OR5P2 | 0.39910974 | 4.50E-06 | 9.60E-05 | **** |
| EDIL3 | 0.39852538 | 1.57E-05 | 0.00023988 | **** |
| SNORA72 | 0.38930299 | 1.11E-05 | 0.00018553 | **** |
| LINC01535 | 0.38822186 | 1.61E-11 | 1.32E-08 | **** |
| LINC00665 | 0.38822186 | 1.61E-11 | 1.32E-08 | **** |
| LAMB1 | 0.38680502 | 2.10E-10 | 8.50E-08 | **** |
| HEY2 | 0.38652217 | 1.17E-08 | 1.45E-06 | **** |
| PXDN | 0.38592589 | 3.53E-09 | 6.25E-07 | **** |
| RNU6-1078P | 0.38569979 | 4.14E-05 | 0.00049757 | **** |
| PCDHB10 | 0.38296335 | 1.15E-08 | 1.43E-06 | **** |
| SCRG1 | 0.38249044 | 4.75E-06 | 9.99E-05 | **** |
| OR2L8 | 0.38194593 | 3.01E-05 | 0.00039231 | **** |
| ZNF711 | 0.38185062 | 2.01E-11 | 1.56E-08 | **** |
| RNU6-1052P | 0.38137616 | 3.59E-06 | 8.05E-05 | **** |
| ST8SIA6 | 0.38050068 | 5.95E-05 | 0.00064706 | **** |
| OBP2B | 0.37934072 | 4.42E-09 | 7.00E-07 | **** |
| RNU6-1079P | 0.37765373 | 1.44E-08 | 1.67E-06 | **** |
| EFHD1 | 0.37444045 | 8.86E-13 | 1.74E-09 | **** |
| MFGE8 | 0.37383908 | 4.49E-12 | 7.16E-09 | **** |
| MMP16 | 0.37267307 | 1.75E-07 | 1.02E-05 | **** |
| RASGRP1 | 0.37199506 | 6.29E-07 | 2.42E-05 | **** |
| NID1 | 0.36872384 | 3.11E-08 | 2.97E-06 | **** |
| ZNF726 | 0.36701721 | 4.75E-08 | 4.18E-06 | **** |
| BAMBI | 0.36282955 | 1.14E-07 | 7.51E-06 | **** |
| COL9A1 | 0.36266435 | 1.94E-06 | 5.13E-05 | **** |
| EIF3D | 0.3626212 | 7.16E-06 | 0.00013305 | **** |
| CBX3 | 0.36237291 | 2.16E-07 | 1.18E-05 | **** |
| VIT | 0.35917076 | 2.69E-07 | 1.35E-05 | **** |
| CEP170 | 0.35889924 | 2.71E-06 | 6.60E-05 | **** |
| ANKRD36 | 0.35699288 | 1.54E-07 | 9.36E-06 | **** |
| SEPTIN4 | 0.35515236 | 6.34E-10 | 1.84E-07 | **** |
| NRCAM | 0.35339438 | 1.27E-06 | 3.82E-05 | **** |
| GUCY1A1 | 0.35163716 | 1.46E-07 | 9.05E-06 | **** |
| MYEF2 | 0.35115042 | 7.92E-09 | 1.08E-06 | **** |
| PRLR | 0.35001727 | 2.77E-06 | 6.71E-05 | **** |
| RNU6-1054P | 0.34965688 | 1.18E-06 | 3.63E-05 | **** |
| RNU6-705P | 0.34965688 | 1.18E-06 | 3.63E-05 | **** |
| RNU6-791P | 0.34965688 | 1.18E-06 | 3.63E-05 | **** |
| RNU6-785P | 0.34965688 | 1.18E-06 | 3.63E-05 | **** |
| RNU6-1100P | 0.34965688 | 1.18E-06 | 3.63E-05 | **** |
| RNU6-1076P | 0.34965688 | 1.18E-06 | 3.63E-05 | **** |
| RNU6-1199P | 0.34965688 | 1.18E-06 | 3.63E-05 | **** |
| RNU6-1118P | 0.34965688 | 1.18E-06 | 3.63E-05 | **** |
| RNU6-1319P | 0.34965688 | 1.18E-06 | 3.63E-05 | **** |
| LINC01002 | 0.34965688 | 1.18E-06 | 3.63E-05 | **** |
| RNU6-241P | 0.34965688 | 1.18E-06 | 3.63E-05 | **** |
| RNU6-860P | 0.34965688 | 1.18E-06 | 3.63E-05 | **** |
| RNU6-447P | 0.34965688 | 1.18E-06 | 3.63E-05 | **** |
| RNU6-177P | 0.34965688 | 1.18E-06 | 3.63E-05 | **** |
| RNU6-355P | 0.34965688 | 1.18E-06 | 3.63E-05 | **** |
| RNU6-1217P | 0.34965688 | 1.18E-06 | 3.63E-05 | **** |
| RNU6-747P | 0.34965688 | 1.18E-06 | 3.63E-05 | **** |
| PCDHB14 | 0.34954243 | 3.27E-06 | 7.52E-05 | **** |
| EPHA7 | 0.34927451 | 4.50E-06 | 9.60E-05 | **** |
| KIT | 0.34901189 | 1.16E-07 | 7.59E-06 | **** |
| TAS2R4 | 0.34823661 | 4.22E-10 | 1.35E-07 | **** |
| ZFHX4 | 0.3480022 | 1.62E-06 | 4.47E-05 | **** |
| OR2AK2 | 0.3465251 | 2.89E-05 | 0.00038034 | **** |
| PDZRN3 | 0.34515216 | 1.50E-10 | 6.70E-08 | **** |
| DLX5 | 0.34514912 | 9.88E-06 | 0.00016981 | **** |
| C8orf44 | 0.34472259 | 3.78E-07 | 1.70E-05 | **** |
| MIR186 | 0.34462851 | 4.34E-05 | 0.00051453 | **** |
| CCDC14 | 0.34443694 | 3.49E-09 | 6.24E-07 | **** |
| SERPINE2 | 0.34213506 | 7.66E-08 | 5.70E-06 | **** |
| RNU4-36P | 0.34037815 | 2.09E-06 | 5.46E-05 | **** |
| OLFM4 | 0.33826757 | 0.01252851 | 0.03638119 | ** |
| ZNF286A-TBC1D26 | 0.33621291 | 1.14E-10 | 5.30E-08 | **** |
| ZNF286B | 0.33621291 | 1.14E-10 | 5.30E-08 | **** |
| ZNF286A | 0.33621291 | 1.14E-10 | 5.30E-08 | **** |
| MPPED2 | 0.33591259 | 4.38E-05 | 0.00051812 | **** |
| BICC1 | 0.33535892 | 1.21E-09 | 2.85E-07 | **** |
| RNU6-943P | 0.33290691 | 0.00046322 | 0.00303529 | *** |
| SYCP2L | 0.33058379 | 3.10E-06 | 7.24E-05 | **** |
| CLUL1 | 0.32732652 | 1.83E-09 | 4.06E-07 | **** |
| USP32P2 | 0.32705686 | 1.95E-09 | 4.28E-07 | **** |
| RNU6-336P | 0.32607899 | 0.00055872 | 0.00345758 | *** |
| RPS24 | 0.32586358 | 0.00069474 | 0.00410215 | *** |
| PTH2R | 0.32564258 | 3.06E-05 | 0.00039785 | **** |
| MTND1P32 | 0.32499485 | 4.74E-08 | 4.18E-06 | **** |
| MTND2P2 | 0.32499485 | 4.74E-08 | 4.18E-06 | **** |
| RAPGEF4 | 0.32472095 | 9.84E-07 | 3.28E-05 | **** |
| RNU6-729P | 0.32239075 | 0.00011925 | 0.00109646 | **** |
| LGR6 | 0.32142186 | 3.17E-07 | 1.51E-05 | **** |
| OR2L2 | 0.32003976 | 0.0001344 | 0.00119606 | **** |
| SNORA62 | 0.31982881 | 2.96E-06 | 7.00E-05 | **** |
| CDK6 | 0.31929487 | 1.30E-08 | 1.60E-06 | **** |
| NPNT | 0.31844368 | 1.14E-08 | 1.43E-06 | **** |
| RNU6-513P | 0.31812206 | 5.30E-08 | 4.52E-06 | **** |
| DPY19L2 | 0.31796999 | 2.91E-09 | 5.76E-07 | **** |
| RNU6-522P | 0.31597276 | 0.00151425 | 0.0072789 | *** |
| SNORD117 | 0.31585319 | 0.00452854 | 0.01672599 | ** |
| GCNT2 | 0.31584477 | 6.87E-08 | 5.34E-06 | **** |
| RNU6-808P | 0.31562835 | 1.59E-05 | 0.00024333 | **** |
| CCDC158 | 0.31558789 | 1.49E-08 | 1.69E-06 | **** |
| TBC1D32 | 0.31409649 | 8.92E-08 | 6.42E-06 | **** |
| SNORD56B | 0.31285921 | 0.00017228 | 0.00144488 | *** |
| SNORD58B | 0.31285568 | 0.0153578 | 0.042747 | ** |
| RPL17 | 0.31285568 | 0.0153578 | 0.042747 | ** |
| LINC00885 | 0.31077339 | 7.87E-12 | 9.68E-09 | **** |
| RPL12P16 | 0.30910188 | 3.06E-07 | 1.47E-05 | **** |
| NBEAL1 | 0.30910188 | 3.06E-07 | 1.47E-05 | **** |
| TMSB15B | 0.30901028 | 7.46E-09 | 1.03E-06 | **** |

**Supplementary Table 7.** ACC gene patient signature**.** List of the 156 genes extrapolated from the intersection of the upregulated genes from publicly available datasets (Andersson, Chowbina, Gao) and the RNA-seq analysis carried out in this study (Cicirò).

| **ACC GENE SIGNATURE** | | | | | |
| --- | --- | --- | --- | --- | --- |
| PNMA8A | SHC4 | ZWILCH | KIF14 | NCAPG2 | BZW2 |
| TMEFF1 | GGH | ATIC | GINS1 | C1GALT1C1L | MTHFD2 |
| ARNT2 | TFAP2A | SCRG1 | WDR12 | MCM3 | BICD1 |
| CHODL | HEY2 | GABRP | PRELP | ARL9 | TOP2A |
| TTK | CENPU | WDFY2 | MYH10 | OLFM2 | ANKRD50 |
| OBP2B | CENPK | HAPLN3 | AADAT | EFNA3 | BCL2 |
| VCAN | CENPE | TM4SF1 | ZBED4 | ZNF286B | CHEK1 |
| AGPAT5 | WDHD1 | FAT1 | NUDT11 | ZNF286A | RASGRP1 |
| C4orf46 | GART | BUB1B | SLC25A15 | CCND1 | TRAM1L1 |
| STK26 | CHML | FAM216A | BUB1 | SGO2 | SLC35F3 |
| PCDHB10 | CCDC138 | PGAP1 | BAMBI | RFC3 | PLK4 |
| CDC7 | NOTCH1 | KPNA2 | ZNF260 | COL11A1 | APBB2 |
| RPP40 | TPST1 | BMPR1B | ZNF257 | VIT | RAD51AP1 |
| SOX4 | PRAME | SCHIP1 | MLC1 | ZNF681 | TRPS1 |
| NETO2 | SERPINH1 | FAM178B | TP53 | ST3GAL4 | APBA2 |
| E2F7 | FLVCR1 | EZH2 | ZNF239 | CTTNBP2NL | SDC2 |
| E2F3 | DDIAS | TTYH1 | PUS7 | ABI2 | SELENOI |
| DTL | DIPK1C | SPARC | ART3 | PCDHB3 | DAPK1 |
| SKA3 | ZFP37 | WEE1 | NCAPG | PCDHB2 | ZNF300 |
| FABP7 | SERPINE2 | TUSC3 | KNTC1 | POLR1B | MOK |
| PLXDC2 | FNDC1 | AFAP1 | PLSCR3 | EFHD1 | FANCI |
| SPPL3 | UBE2T | PRKDC | CDC25B | MYB | NCKAP5 |
| BRCA2 | LAMB1 | ANLN | ARHGEF9 | GUCY1B1 | MTHFD1L |
| NUF2 | TEX14 | KIF23 | LIMK2 | CHD1L | CDC42EP3 |
| VTCN1 | POLE2 | EN1 | EPHX4 | KIAA0895 | RPGRIP1L |
| ZNF492 | HORMAD1 | KIF15 | GLMN | GUCY1A1 | MIA |
