# Supplementary material for: The mitotic checkpoint kinase BUB1 is a direct and actionable target of MYB in adenoid cystic carcinoma
Source: FEBS Lett. 2023 Dec 27;598(2):252–65. doi: 10.1002/1873-3468.14786 (PMC11774229; doi:10.1002/1873-3468.14786)
Supplement: Supplementary file 3 — Data S1. Supplementary methods and references. [file FEB2-598-252-s002.docx]

**Supplementary methods and references**

Apoptosis assay

For apoptosis assays, cells were seeded in white-walled 96-well luminometer plates with or without the BUB1 inhibitor. Caspase-Glo 3/7 Reagent (Promega) was added in a proportion 1:2 to the volume of medium in the well and gently mixed by pipetting. Plates were incubated for 1 hour at room temperature. Luminescence emission was measured on a Victor-3 multilabel reader (PerkinElmer).

## Luciferase vectors construction and luciferase assay

A segment of 500 bp of the *BUB1* promoter upstream the transcription start site (TSS) (codon ATG, position 110677995) was cloned into the pGL3-Basic backbone vector upstream the luciferase gene using KpnI and XhoI as restriction enzymes at the 5’ and 3’ ends, respectively. The pGL3-BUB1 Mut plasmid contains mutations of the four canonical MYB binding sequences present in the 500bp promoter segment (**Supplementary figure 1**). Gene synthesis and cloning was performed by Biomatik Corporation.

For luciferase assays, 5x10^4^/well of MCF10A cells were seeded in 24-well plates. The next day, cells were co-transfected with 0.5 μg of pGL3-BUB1 (WT or Mut) and pLXSN-MYB (or empty vector) plasmids[1]. pRenilla luciferase vector was used to control transfection efficiency. Luciferase activity was detected with the Dual-Luciferase Reporter Assay System (Promega).

Transfection of siRNAs

ACC cells were transfected with 50 nM of Stealth siRNAs targeting *MYB* (HSS106819, HSS106821) or control siRNAs, using the Lipofectamine RNAiMAX transfection reagent (Thermo Fisher Scientific) in antibiotic-free medium and maintained for 48 hours.

Real-time quantitative polymerase chain reaction (RT-qPCR)

Total RNA was extracted from cultured cells using the Monarch Total RNA Miniprep Kit (New England BioLabs) according to the manufacturer’s instructions. RNA was reverse-transcribed into complementary DNA (cDNA) using the High-Capacity RNA- to-cDNA Kit (Applied Biosystems). For MCF10A cells, qPCR was performed according to the FastGene 2x IC Green Universal qPCR Mix (fluorescein) (Nippon Genetics Düren) protocol. The primers used were: *MYB*, (forward) GGGAACAGATGGGCAGAAATCG and (reverse) GCTGGCTTTTGAAGACTCCTGC; *BUB1*, (forward) GCTCTGTCAGCAGACTTCCTTC and (reverse) CAGCAGATGTGAAGTCTCCTGG; *GAPDH* (forward) GTCTCCTCTGACTTCAACAGCG and (reverse) ACCACCCTGTTGCTGTAGCCAA. For ACC cells, qPCR was carried out using TaqMan Gene Expression Assays (Applied Biosystems) for *MYB* (Hs00920556_m1*) and *BUB1* (Hs01557695_m1). *UBC496* (Hs01871556_s1) was used as the reference gene.

Western blot

Cells were rinsed with ice-cold 1X PBS (Gibco) supplemented with 1X cOmplete, EDTA-free Protease Inhibitor Cocktail (Roche). Direct lysis was performed by adding 1X Laemmli buffer (approximatively 60 µL/1 x 10^6^ cells) to the cells and mechanically scraping them from the plate. C-Myb Antibody (D-7): sc-74512 (Santa Cruz Biotechnology) was used at 1: 500 dilution; GAPDH Monoclonal antibody (Proteintech) was used at 1:1000 dilution; anti-mouse IgG, HRP-linked Antibody #7076 (Cell Signaling Technology) was used at a dilution 1: 10,000.

Chromatin immunoprecipitation (ChIP) and ChIP sequencing

Chromatin immunoprecipitations (ChIP) were performed with cross-linked chromatin from ACCX11 cells using SimpleChIP Enzymatic Chromatin IP Kit (Magnetic Beads) (Cell Signaling Technology) following manufacturer’s instructions. Anti-c-Myb (phospho S11) [EP769Y] (ab45150) (Abcam) antibody, was used at a dilution of 1:50. Chromatin immunoprecipitation sequencing (ChIP-seq) (including library construction) was performed by Novogene. Libraries were constructed using Illumina Sequencing PE150 library preparation. Sequencing was performed at 20M reads per sample.

RNA sequencing

RNA sequencing (including library construction and sequencing) was performed by Novogene. Libraries were constructed using Illumina Sequencing PE150 library preparation with Ribozero rRNA depletion. Sequencing was performed on Novaseq 6000 platform, at 20M paired-end reads per sample.

Bioinformatic analyses

Fastq RNA-seq raw reads were analysed with the open-source software package of the Tuxedo Suite. Tophat2 with bowtie2 were used to map paired-end read [2, 3]. The *Homo sapiens* genome build GRCh38 was used as reference. GENCODE38 was used as the reference human genome annotation[4]. Aligned reads were filtered by quality using samtools with a minimum selection threshold set at 30 [5]. Transcript assembly and quantification was achieved using HTSeq 2.0 [6]. Differential expression between sample and control was performed by collapsing technical replicates for each condition and the use of the DESeq2 tool in R library v 1.32.0 [7].

ChIP-seq data in the form of fastq files were mapped to the Homo sapiens GRCh38 reference genome using bowtie2. Reads were filtered by a standard quality threshold of 30 using samtools[5]. MACS was used to call peaks with a p value threshold of 0.01 by comparison with the input control. True peaks were selected when two of three replicates overlap in the same sequence. Binding motif analysis and peak visualisation was performed on Integrative Genomics Viewer (IGV).

GO analysis was performed using the online tool ExpressAnalyst (www.expressanalyst.ca) and the PANTHER Biological Process (BP) repositor. GO and pathway terms were filtered by false discovery rate (FDR) ≤ 0.1.

GSEA was performed on the GSEA software v 4.2.3 by inputting RNA-seq values in the values of counts generated by HTSeq 2.0 against a custom gene signature[6]. Standard GSEA settings were employed: 1000 permutations, gene set, and Signal2Noise metric.

1. Sala A, Bellon T, Melotti P, Peschle C & Calabretta B (1995) Inhibition of erythro-myeloid differentiation by constitutive expression of a DNA binding-deficient c-myb mutant: implication for c-myb function. Blood 86, 3404-3412.

2. Kim D, Pertea G, Trapnell C, Pimentel H, Kelley R & Salzberg SL (2013) TopHat2: accurate alignment of transcriptomes in the presence of insertions, deletions and gene fusions. Genome Biol 14, R36, doi: 10.1186/gb-2013-14-4-r36.

3. Langmead B & Salzberg SL (2012) Fast gapped-read alignment with Bowtie 2. Nat Methods 9, 357-359, doi: 10.1038/nmeth.1923.

4. Frankish A, Diekhans M, Ferreira AM, Johnson R, Jungreis I, Loveland J, Mudge JM, Sisu C, Wright J, Armstrong J, Barnes I, Berry A, Bignell A, Carbonell Sala S, Chrast J, Cunningham F, Di Domenico T, Donaldson S, Fiddes IT, Garcia Giron C, Gonzalez JM, Grego T, Hardy M, Hourlier T, Hunt T, Izuogu OG, Lagarde J, Martin FJ, Martinez L, Mohanan S, Muir P, Navarro FCP, Parker A, Pei B, Pozo F, Ruffier M, Schmitt BM, Stapleton E, Suner MM, Sycheva I, Uszczynska-Ratajczak B, Xu J, Yates A, Zerbino D, Zhang Y, Aken B, Choudhary JS, Gerstein M, Guigo R, Hubbard TJP, Kellis M, Paten B, Reymond A, Tress ML & Flicek P (2019) GENCODE reference annotation for the human and mouse genomes. Nucleic Acids Res 47, D766-D773, doi: 10.1093/nar/gky955.

5. Li H, Handsaker B, Wysoker A, Fennell T, Ruan J, Homer N, Marth G, Abecasis G, Durbin R & Genome Project Data Processing S (2009) The Sequence Alignment/Map format and SAMtools. Bioinformatics 25, 2078-2079, doi: 10.1093/bioinformatics/btp352.

6. Putri GH, Anders S, Pyl PT, Pimanda JE & Zanini F (2022) Analysing high-throughput sequencing data in Python with HTSeq 2.0. Bioinformatics 38, 2943-2945, doi: 10.1093/bioinformatics/btac166.

7. Love MI, Huber W & Anders S (2014) Moderated estimation of fold change and dispersion for RNA-seq data with DESeq2. Genome Biol 15, 550, doi: 10.1186/s13059-014-0550-8.

8. Andersson MK, Afshari MK, Andren Y, Wick MJ & Stenman G (2017) Targeting the Oncogenic Transcriptional Regulator MYB in Adenoid Cystic Carcinoma by Inhibition of IGF1R/AKT Signaling. J Natl Cancer Inst 109, doi: 10.1093/jnci/djx017.

9. Gao R, Cao C, Zhang M, Lopez MC, Yan Y, Chen Z, Mitani Y, Zhang L, Zajac-Kaye M, Liu B, Wu L, Renne R, Baker HV, El-Naggar A & Kaye FJ (2014) A unifying gene signature for adenoid cystic cancer identifies parallel MYB-dependent and MYB-independent therapeutic targets. Oncotarget 5, 12528-12542, doi: 10.18632/oncotarget.2985.
